# Transcriptomic identification of candidate genes involved in sunflower responses to chilling and salt stresses based on cDNA microarray analysis

**DOI:** 10.1186/1471-2229-8-11

**Published:** 2008-01-26

**Authors:** Paula Fernandez, Julio Di Rienzo, Luis Fernandez, H Esteban Hopp, Norma Paniego, Ruth A Heinz

**Affiliations:** 1 Instituto de Biotecnología, CICVyA, INTA Castelar, Las Cabañas y Los Reseros, (B1712WAA) Castelar, Provincia de Buenos Aires, Argentina; 2 Cátedra de Estadística y Biometría, Facultad de Ciencias Agrarias, Universidad Nacional de Córdoba, Córdoba, Argentina; 3 Facultad de Ciencias Exactas y Naturales, Universidad de Buenos Aires, Buenos Aires, Argentina

## Abstract

**Background:**

Considering that sunflower production is expanding to arid regions, tolerance to abiotic stresses as drought, low temperatures and salinity arises as one of the main constrains nowadays. Differential organ-specific sunflower ESTs (expressed sequence tags) were previously generated by a subtractive hybridization method that included a considerable number of putative abiotic stress associated sequences. The objective of this work is to analyze concerted gene expression profiles of organ-specific ESTs by fluorescence microarray assay, in response to high sodium chloride concentration and chilling treatments with the aim to identify and follow up candidate genes for early responses to abiotic stress in sunflower.

**Results:**

Abiotic-related expressed genes were the target of this characterization through a gene expression analysis using an organ-specific cDNA fluorescence microarray approach in response to high salinity and low temperatures. The experiment included three independent replicates from leaf samples. We analyzed 317 unigenes previously isolated from differential organ-specific cDNA libraries from leaf, stem and flower at R1 and R4 developmental stage. A statistical analysis based on mean comparison by ANOVA and ordination by Principal Component Analysis allowed the detection of 80 candidate genes for either salinity and/or chilling stresses. Out of them, 50 genes were up or down regulated under both stresses, supporting common regulatory mechanisms and general responses to chilling and salinity. Interestingly 15 and 12 sequences were up regulated or down regulated specifically in one stress but not in the other, respectively. These genes are potentially involved in different regulatory mechanisms including transcription/translation/protein degradation/protein folding/ROS production or ROS-scavenging. Differential gene expression patterns were confirmed by qRT-PCR for 12.5% of the microarray candidate sequences.

**Conclusion:**

Eighty genes isolated from organ-specific cDNA libraries were identified as candidate genes for sunflower early response to low temperatures and salinity. Microarray profiling of chilling and NaCl-treated sunflower leaves revealed dynamic changes in transcript abundance, including transcription factors, defense/stress related proteins, and effectors of homeostasis, all of which highlight the complexity of both stress responses. This study not only allowed the identification of common transcriptional changes to both stress conditions but also lead to the detection of stress-specific genes not previously reported in sunflower. This is the first organ-specific cDNA fluorescence microarray study addressing a simultaneous evaluation of concerted transcriptional changes in response to chilling and salinity stress in cultivated sunflower.

## Background

Sunflower (*Helianthus annuus *L.) is the third most important source of edible vegetable oil worldwide which is also thought to become an efficient source of biodiesel (Sunflower Statistics NSA 2007, USA) [1]. Considering that sunflower production is expanding to arid regions in the Mediterranean area, North America, India and Argentina, tolerance to drought and salinity arises as important issues for breeding programs [2-4]. In addition, requirements of early sow to maximize the growing season and to escape to drought stress have increased the need of better chilling tolerance, particularly at early stages of development. Molecular mechanisms involved in response to these stresses have been extensively studied in model species like *Arabidopsis thaliana *[5-7] and in important crop species like rice [8]. The expression of a number of plant genes is regulated by abiotic environmental stresses including drought, high salinity and cold [9-12]. Transcriptome analysis using microarrays have proven to be a powerful tool for discovery of many stressed-induced genes involved in stress response and tolerance. Macro and microarray studies of abiotic stress responses in *Arabidopsis *and *Oryza sativa *allowed the identification of genes involving both functional and regulatory proteins [6,8,13-23]. The first group comprises membrane transporters and water channel proteins, key enzymes for osmolite biosynthesis; detoxification enzymes and macromolecules protection proteins. The second group comprises transcription factors (TFs) (i.e. bZIP, MYC, MYB, CREB/CBF, HD-ZIP), protein kinases and proteinases involved in the regulation of signal transduction and gene expression. These regulatory systems have been reported either as dependent or independent on abscisic acid (ABA) which indicate the existence of complex regulatory mechanisms between perception of abiotic stress signals and gene expression [20,21].

Cross talk signaling cascades among drought, cold and salinity has been reported for *A. thaliana *and large number of stress-inducible genes were isolated and characterized including osmotic response genes as rd22BP1, AtMYBB2, DREB1A and DREB2A, signaling molecules that activates effectors as SOS3 (Ca^2+ ^binding protein), SOS2 (Ca^2+ ^dependent kinase), SOS1 (Na^+^/H^+ ^membrane antiporter) [8,22]. Regarding responses to low temperatures, cold-induced genes were reported in many species as lucerne [23], *Arabidopsis *[24-26], barley [27] and wheat [28,29]. Many of these genes encode for proteins of unknown function, being some of them described as LEA proteins (Late Embryogenesis Abundant) [30]. Other cold-resistance genes (COR) as LT1, KIN, RD and ERD have been isolated mainly from *Arabidopsis*. These genes present the CRT/DRE (C-repeat/dehydratation-responsive elements) in the promoter region that bind CBF and DREB (C-repeat binding factors/dehydration-responsive elements binding proteins) TFs. While *cis *elements in cold response genes bind DREB1/CBF TFs, regulatory regions of drought response genes bind TFs belonging to DREB 2 type protein [5,31-33].

As mentioned, tolerance to a combination of different abiotic stresses is a well-known breeding target for sunflower as well as for other crops. Studies of simultaneous stress exposure were documented in various plant systems [8,13,34-41]. Nevertheless, little is known about the comparative molecular mechanisms underlying the acclimation responses of plants to a combination of different stresses [42].

Gene expression databases are increasing exponentially and the resulting information is stored and classified. While powerful software algorithms allow structural sequence similarity comparisons between species, difficulties arise to predict molecular function based on comparisons with homologous genes identified as determinant for a specific trait in different species. Identification of true orthologous among species is a powerful tool for candidate gene detection, particularly when comparing species having their full genome sequenced and those based on EST sequencing projects. In the case of *Asteraceae *species (including sunflower), only small syntenic fragments with *Arabidopsis *could be identified and their evolution involving major chromosomal rearrangements makes orthologous gene pairs difficult to identify [43]. Even for other plant taxa comparative functional transcriptomic studies among crop plant genomes is relatively scarce [35]. A microarray analysis in *A. thaliana *to identify simultaneously conserved and differentially expressed genes in oat, poplar and *Euphorbia esula L*. ("leafy spurge") was recently reported [44].

Sunflower was described as normally susceptible to low temperatures and salinity [45,46], however, available information on gene expression in response to abiotic stresses are still limited to few studies [35,45-49]. Recently the detection of a large number of down-regulated genes in plants exposed to extensive periods of low temperatures was reported [35], indicating that acclimation to chilling temperatures does not occur in sunflower. Meanwhile, transcriptional profiles in drought-tolerant and non-tolerant sunflower genotypes in response to water-stress allowed the identification of differential gene expression related to amino acid and carbohydrate metabolisms and signal-transduction processes [49]. In this work we report for the first time a concerted study on gene expression in early responses to chilling and salinity using a fluorescence microarray assay based on organ-specific unigenes in sunflower. The aim of this work was to detect candidate genes associated to regulatory and stress-response pathways common to both stresses and at the same time identify those genes exclusively expressed in response to each kind of stress conditions.

## Results and Discussion

### Data analysis and accuracy of biological replicates

Differential expression of organ-specific sunflower sequences previously obtained by suppression subtractive hybridisation (SSH) [50] was evaluated with regard to the response to chilling and salt stresses using cDNA fluorescence microarray hybridization followed by Northern blot and qRT-PCR validation. Thus, three biological replicates were evaluated for chilling and salinity stresses as well as for control plants. The stress treatments were designed considering that sunflower has been described as normally susceptible to low temperatures and salinity [45,46]. Regarding chilling tolerance, there is only one report studying sunflower response to low temperatures [35]. However, in that study only long-term acclimatizing was evaluated, thus meaning that detected changes in gene expression reflect mainly plant metabolism adaptation to grow under suboptimal conditions more than short term responses to chilling. No previous studies on concerted gene expression of cultivated sunflower to salinity were reported before. In the present work, an early response to chilling and salinity is evaluated in order to detect early transcriptional changes in genes induced at the onset of the tolerance process.

**Figure 1:**
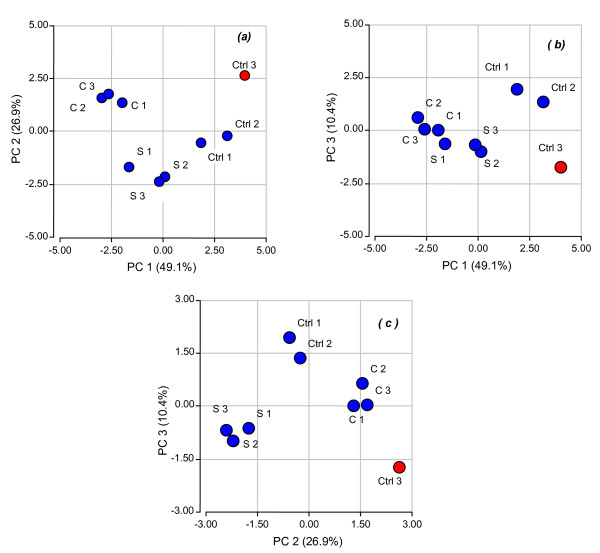
**Principal component analysis (PCA) applied to the gene expression matrix**. Graphical representation of three biological replicates for abiotic stress treatments: control (Ctrl), cold stress (C) and salinity stress (S).

A first step analysis was performed to determine the accuracy and reproducibility of these biological treatments by Principal Component Analysis (PCA) applied to the gene expression matrix (Figure 1). The resulting analysis showed that biological samples of plants that were stressed either with saline or chilling treatments showed expected changes in their general expression parameters when compared to the controls. However, this analysis indicated that one of the biological control replicates (Ctrl 3) displayed a non-typical performance within the control group (Figure 1). Graphical representation of biological replicates in the space spanned by principal components 1,2; 1,3 and 2,3 is shown in Figure 1a, b and 1c, respectively. It is clearly shown that Ctrl3 differed significantly from the other control replicates and was discarded for further analysis.

### Candidate gene selection

#### Data normalization - Normalization within microarrays

**Figure 2:**
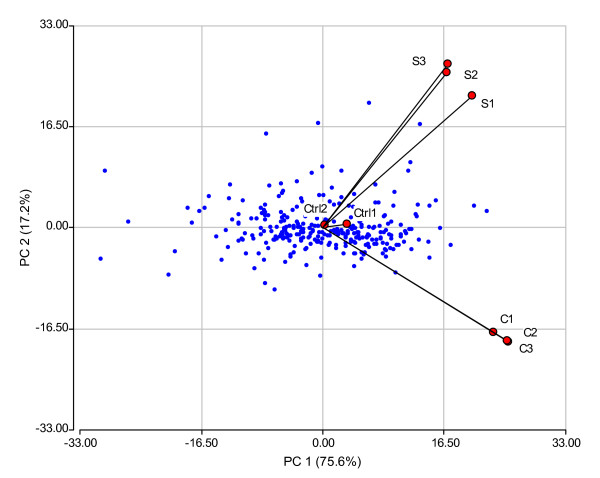
**Bi-plot A**. Biplot showing 287 genes whose expression levels were studied in three treatments: control (Ctrl), cold (C) and salinity (S). The ordination was obtained taking into account the three (two in case of Ctrl) independent biological replicates.

Relationship between genes and treatments as well as the magnitude of their association can be clearly visualized in the bi-plot generated by the first two principal components of the expression matrix, with rows and columns representing genes and treatments (control, cold and salinity) replicates, respectively (Figure 2). In this plot, genes are displayed as points on the plane while treatment replicates are represented by vectors rooted to the origin (centre of the plot). These vectors describe directions along which genes can be ordered according to treatment response. Differentially expressed genes on a specific treatment have a large projection along the axis defined by the direction vector representing that treatment. Replicates from a given treatment have similar orientation and small angles among them, which emphasizes the identity of the treatments (Figure 2). Genes displayed far from the centre of the plot along the "cold vectors" correspond to those over expressed in that treatment, whereas those following the "salinity vectors" correspond to genes over expressed under saline medium. On the other hand, genes placed at the opposite direction of the previous ones are genes sub-expressed under those conditions. Vectors representing the control replicates are shorter than vectors representing stress replicates. This finding supports the fact that a pool of control plants was used as a reference through the hybridization experiments and the expected log fold change for the control treatment should be zero for every gene.

Principal axis 1, which retained 75.6% of the total variability, sorts genes according their fold change in over (on the right) and under (on the left) expression conditions independently of the stress condition applied. Principal axis 2 retaining 17.2% of total variability emphasizes the differences between cold and salinity treatments.

**Figure 3:**
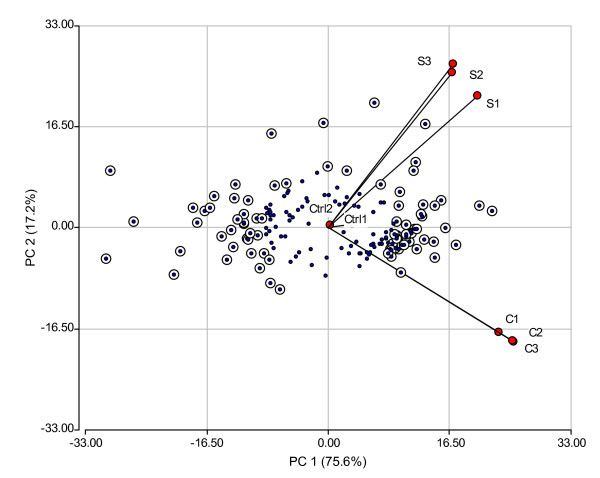
**Bi-plot B**. Biplot of the expression matrix showing only those genes having p-values lower than 0.05 in the F-test. Genes with distance-to-the-origin greater than the 70^th ^percentile of the distance-to-the-origin distribution are shown as dotted circles. The circled dots represent the 80 differentially expressed genes identified as differentially expressed among the evaluated treatments: control (Ctrl), cold (C) and salinity (S). Solid dots represent putative false positive genes.

Although bi-plot representation revealed a clear picture of the high quality of microarray data, it is not by itself an inferential technique. Analysis of variance takes into account variability between replicates to decide on the significance of differential expression [35]. In order to select differentially expressed genes a two step procedure was used. First an analysis of variance for every gene was performed and only those genes having p-values lower than 5% were retained for complementary analysis. It is known that using the raw p-value as a selection criteria results in a large number of false discoveries. This was a matter of concern because nearly 50% of all evaluated ESTs were significant at a 5% significance level. At this point we decided to use information provided by the ordination technique to filter genes and reduce the rate of false discoveries. The rationality behind this procedure is that the farther the gene is to the center of the plot, the larger the fold change expression level. Under this assumption, those genes located in the periphery of the bi-plot should be the most dramatically involved in the responses to stress. The calculation of the distance-to-the-origin in the space spanned by the first two principal components is the first step that serves as a scoring system to rank differentially expressed genes. Thus, within those genes retained by the p-value criteria we kept those that were at a distance-to-the-origin above the percentile 70^th ^of the distance-to-the-origin distribution (Figure 3). This cut-off criterion was selected taking into account that EST T411 (contig of the EST T111, AN: BU671801) was already experimentally validated as differentially expressed by Northern-blot and qRT-PCR and that its position in the distance-to-the-origin distribution corresponds to the 70^th ^percentile (Figure 3). Therefore, 80 genes differentially expressed were picked as candidate genes for early response to low temperatures and salinity (see Additional file 1).

### Microarray validations

In order to experimentally validate differentially expressed genes derived from microarray analysis, Northern blot analysis and qRT-PCR were performed for candidate gene T411 [GenBank: BU671801], not only to validate the differential expression of this gene but also to set the cut-off significance criteria in the Bi-plot analysis within those genes retained by the p value criteria. This gene is up-regulated under cold and salinity stresses (see Additional files 2 and 3) and its position in the distance-to-the-origin distribution corresponds to the 70^th ^percentile, as previously described.

A total of ten candidate genes were validated by qRT-PCR: EF127 [GenBank: BU671885], EF264 [GenBank: BU671886], EF502 [GenBank: BU671910], F171 [GenBank: BU671987], F379 [GenBank: BU671983], F443 [GenBank: BU671999], F455 [GenBank: BU672004], H360 [GenBank: BU672086], T124 [GenBank: BU671806] and T411 [GenBank: BU671801] using specif oligonucleotides designed for each candidate gene (Table 1). Three biological replicates of each reaction derived from independent cDNA synthesis were performed and actin sequence from sunflower [GenBank: AAF82805] was used as an internal control to normalize gene expression level. Quantification of the relative changes in gene expression was performed using the 2^-ΔΔCT ^method as described by Livak and Schmittgen [51]. Comparison of the results from real-time RT-PCR with those from microarray analysis revealed similar patterns of expression. Pearson's correlation coefficient between cDNA microarray and qRT-PCR fold changes was r = 0.60 (p = 0.0054) (Table 2) (see Additional file 4). More than 10% of the candidate genes detected by microarray assay in the present study were validated by qRT-PCR. Considering that average validation percentage is usually below 5% out of total candidate genes for reported cDNA microarrays studies [35,49,52] the number of validated genes in this work is highly representative of the transcription profile patterns detected by the microarray technology (12.5% out of total of differentially expressed genes) (Table 2). In all cases, observed transcriptional changes confirmed the results of microarray analysis.

**Table 1:** Oligonucleotides used for qRT-PCR validations

**GenBank (dbEST) Accession Number**	**EST/Gene name**	**Forward primer 5'-3'**	**Reverse primer 3'-5'**
BU671885	EF127	GCATTGGGCAGATCTTGTTT	GTCCCCTTTGGAGGCAGTA
BU671886	EF264	GGAGCTTGAGGATGCGATAC	GAAACGTAAAGCCCCGATAA
BU671910	EF502	TGATCCATCAATCTCCGTCTT	TGTAGGTGCATGGAACAAGC
BU671987	F171	AAAGGATCAGTCGCTGCTGT	GCTTTTCCAAGATTGCATCC
BU671927	F126	CAAAATGCAACGACCCATTA	TCTGTACGCCCTCATGTTCA
BU671928	F231	CAACAAAAGCAGACGCTGAA	AGCATGTGGTGTTTGGACAG
BU671983	F379	CAGCCCGGAGAGGTTTAACT	GGCAGGTACAGAATCGGCTA
BU671999	F443	AATCCCATCAATCCCCACTT	GTTTCCACCCCTTCCATTTT
BU672004	F455	GCCGAGGTACAAACTGGAGA	TGAGCATGATCTGAATATCTTGAA
BU672026	F543	ACGGAAGCGTTGTTTGGTAA	TCAACATCCCACAGAAACGA
BU672017	F550	CAGAGACGTTCTTGCGTTGA	CGCACACAACAAAGAAATGG
BU672042	F557	CGCAATTGCTATTGATGGAA	ACACCGGTATGGTTGATGCT
BU672056	H110	ACGCGAGTCGGTTGTTTTAT	TCATTTTCTCCACCCATGGTA
BU672069	H123	GGCAGGTACCAGGGGTTATT	GAGGTTCATTCCGTCGTTGT
BU672102	H136	TTTGCAAGGATGAATGGTGA	GTGACCCGAACTCCTTGGTA
BU672086	H360	GGCAGCCAATCCTCTTGATA	CGACTCCGCCAAATACAGAT
BU672090	H385	TTCAGCCCGGAAAGAATATG	AACTTTGCAGTGGGACCATC
BU671806	T124	GGAACACCGTGAAGGATGAG	GGCAGGTACATCTTGGCCAAT
BU671875	T283	CTCACGAAAGCTTCCTGCTT	GCAGGTACTCGGTTTGTTCC
BU671843	T340	AAGACGGTGGATTTGAGGTG	AACCTTTGCCTGCTTTCTCA
BU671801	T411	GGCAAGGGAAAACACCACTA	TGTTGAGGTGTGGCTCTGTC
AAF82805	*sunflower *actin	AGGGCGGTCTTTCCAAGTAT	ACATACATGGCGGGAACATT

**Table 2:** Comparison of gene expression levels obtained by cDNA microarray and qRT-PCR analysis for 10 differentially expressed genes

**GenBank (dbEST) Accession Number**	**EST/Gene Name**	**Fold change qRT-PCR**				**Fold change microarray**			
		
		**Cold**		**Salinity**		**Cold**		**Salinity**	
BU671885	EF127	4.6100	↑	5.6900	↑	1.7590	↑	1.7430	↑
BU671886	EF264	14.1000	↑	88.1500	↑	0.2670	↑	1.1620	↑
BU671910	EF502	4.8000	↑	-2.1200	↓	1.1010	↑	-1.1410	↓
BU671987	F171	-0.8000	↓	6.2500	↑	-1.7970	↓	1.6890	↑
BU671983	F379	112.1500	↑	0.0280	↑	1.3060	↑	1.1190	↑
BU671999	F443	-140.3200	↓	-110.1200	↓	-1.1790	↓	-1.1030	↓
BU672004	F455	48.1200	↑	82.5600	↑	1.5030	↑	1.3230	↑
BU672086	H360	47.4000	↑	3.8500	↑	1.8140	↑	1.2460	↑
BU671806	T124	13.5000	↑	22.3500	↑	1.3550	↑	1.5200	↑
BU671801	T411	12.4200	↑	2.3800	↑	1.4980	↑	1.2820	↑

### Differences in transcriptional changes detected in organ-specific cDNA libraries

**Figure 4:**
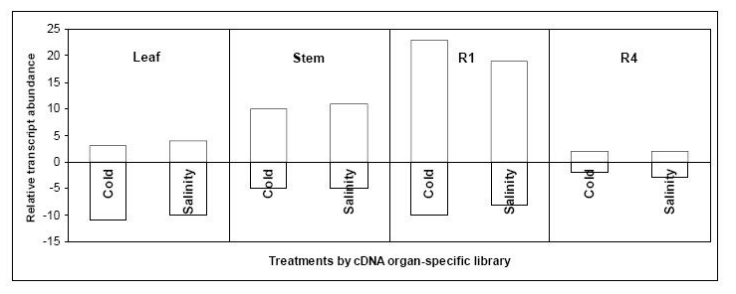
**Profile of gene expression by organ-specific cDNA library**. Transcriptional changes of the 80 differential genes were evaluated according to the organ-specific library from which there were originally isolated. Most of the genes isolated from the leaf cDNA library show a decrease in transcript abundance while genes isolated from R4 library showed an inverse pattern under salt and cold stresses.

Differences in gene expression were analyzed according to the differential organ-specific cDNA library from which they were isolated as described before [50] (Figure 4). Leaf library derived genes (comprising differential sequences of leaf arrested with R4 flower bud) were mostly down-regulated in response to either stress condition, whereas a large number of genes derived from an R4 flower developmental stage and stem libraries (representing differential sequences of flower bud and stem arrested both with leaf, respectively) showed an up-regulation transcriptional pattern. Considering that transcriptional changes in response to salt and chilling stresses are evaluated here in leaf tissue, these results confirmed the efficiency of SSH technique in the generation of the differential organ specific libraries [50]. ESTs from leaf library correspond to genes that are highly expressed in control conditions while the opposite situation takes place with ESTs isolated from the flower and stem libraries. Thus, the majority of the genes from the leaf library evaluated in this assay were down regulated in response to stress conditions while genes derived from stem and R4 flower bud libraries, representing genes at a lower expression level in control leaf, appeared up-regulated in these assays. The set of genes evaluated in the present study is composed mainly by genes that are either at high expression levels in control leaves (those from leaf library) or either genes that are at low expression level in control leaves (those from stem and flower bud libraries). Genes with similar transcription levels in different plant organs under control conditions are low represented in this array. These results also explained the large transcription change/transcription unchanged ratio detected in these assays, considering that 27.8% (80/287) of the evaluated genes appeared down or up-regulated in either one or the other stress condition, when compared to microarray analysis derived from non-subtractive cDNA libraries [21].

### Differences between cold and salinity treatments

Specific gene expression patterns in plants exposed to different treatments are evidenced when profile graphs by gene are analyzed (see Additional file 2). Drought, salinity and cold stress reduce water availability decreasing cell water potential. In order to avoid dehydration, plant responses include solute accumulation, cell wall components modification, and synthesis of protective proteins, avoiding or repairing cellular damage [53]. The activation of these responses requires a complex signaling network being many of them shared by various abiotic stresses as those involving the DREB/CBF pathway [9,54] and other ones typically from a determined abiotic stress [7,55].

Although *heatmaps *are largely used in microarray analysis as a tool to visualize and detect differentially expressed genes [56] (see Additional file 3), an alternative and more reliable tool is the evaluation of individual gene transcription profile (see Additional file 2). Indeed, several reports on the usefulness of both methodologies to analyze microarray results have been recently published [57].

The availability of microarray technology allowing the comparison of large numbers of genes that are regulated under certain condition represents a powerful tool for many researchers. However, caution should be exercised when interpreting the outcome results. For example, salt stress treatment results in a rapid decline in photosynthesis within minutes [37]. Therefore, genes that are regulated by photosynthetic activities maybe affected but they are not regulated by salt stress *per se*. In another study the expression of 150 genes in response to wounding and insect feeding was carried out [58], finding that some of the responding genes, for which the inducing stimulus was unclear in these processes, were also characterized as induced by drought [59]. Thus, several factors could contribute to a complex pattern of transcript levels in which the interplay of stimuli that control gene expression can override each another. The stress intensity and the time course of gene induction are also important factors to be considered [59]. The primary and the secondary stresses may induce genes in different time frames.

In *Arabidopsis *studies on concerted genes expression revealed a large number of cold- acclimation and freezing tolerance genes [6,60-62], but there are only few studies on acclimation to chilling temperature [63]. The ability of sunflower plants to gain a frost tolerance after exposure to a period of low temperature is still poorly understood. Although, recent results suggest that sunflower plants are non-acclimating plants under two conditions of low-term long-temperature at 15°C and 7°C [35]. Thus, in the present study the response of sunflower to low temperatures has been focused at primary responses of young plants to a 24 hs treatment at 10°C with the aim to detect regulatory mechanisms induced at this early stage. Tolerance to low temperatures arise as an important trait considering that sunflower productive area is expanding to marginal regions with suboptimal growing conditions and the increasing requirement of early sow to maximize the growing season in many countries. Regarding tolerance to high NaCl contents, there is not much information available for sunflower but expansion of crops to more arid regions is associated to an increasing problem of soil salinity.

**Figure 5:**
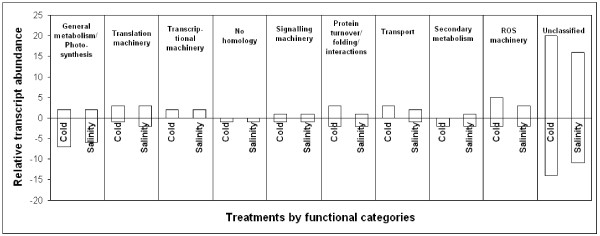
**Profile of gene expression by putative functional category**. The number and direction of transcriptional changes of the 80 differential genes under cold and salinity stresses are presented by functional categories.

The 80 sequences that showed changes in transcriptional levels in response to salt and cold conditions were classified by putative functionality according to best hits on sequence similarity analysis based on BLAST algorithms and GO terminology (see Additional file 1) [64]. Gene expression profile by clustering analysis using *heatmap *representation (see Additional file 3) allowed the detection of gene patterns among treatments (chilling, salinity and control) confirmed by individual gene transcription profiles (see Additional file 2). Out of the 80 candidate, 50 genes were either up or down regulated under both abiotic stresses, thus supporting common regulatory mechanisms and general responses to low temperature and salinity. Fifteen and 12 sequences were either up or down-regulated respectively in a stress-specific manner under chilling or salinity. Finally, 3 genes showed inverted pattern of expression (F171, T107, E502) [GenBank: BU671987] [GenBank: BU671799] [GenBank: BU671910] and 39 differential ESTs correspond to genes with unknown predicted function [64]. The number of genes that were either up or down regulated under salt or chilling stresses are showed in Figure 5 grouped by assigned molecular and/or processes function. Changes in transcriptional patterns in response to chilling and salinity stresses are discussed below according to their predicted functionality classes (see Additional file 1) based on the Gene Ontology [65]. Those accessions without GO-term association were included in a particular category by means of a manual procedure.

#### Central metabolism/Photosynthesis

Low-temperature exposure in combination with high irradiance causes rapid inhibition of photosynthesis in a broad range of plants including tomato, cucumber and maize. Several elements contributing to this inhibition have been identified [66]. Damage to the reducing side of photosystem II is well documented [67,68] and, for moderately sensitive species such as maize, it may be the major cause of impaired whole plant photosynthesis following chilling. However, in the most chill-sensitive species, such as tomato, impaired reductive activation of the stromal biphosphatases appears to be the dominating factor limiting carbon assimilation following chilling in the light [69]. Low temperature at night can also cause severe reductions in CO_2 _fixation on the day after chilling. In this work, genes that encode products with predicted functions related to energy metabolism were down-regulated under both stresses in sunflower. Among them, many genes potentially encoding components involved in photosynthesis, such as photosystem proteins, chlorophyll-binding proteins, Rubisco and light harvesting proteins showed differential expression patterns in this study. Considering sunflower as a medium-tolerant plant to chilling sensitivity [45], it has been suggested that decreasing energy metabolism is one of the cellular processes associated with the sunflower response to low temperatures [68]. Here, we show that this process is not only down-regulated under chilling stress but also under salinity stress in *H. annuus*. Down-regulation of fructose-1,6 biphosfatase under drought stress in sunflower was reported as mainly associated to the stomata closure produced during water deficit [69]. Yet, it was observed that NaCl reduces photosynthetic activity in *Phaseolus vulgaris *independent of stomata closure and by reducing the RuBP pool size through an effect on the RuBP regeneration potential [70]. Thus, decline in photosynthesis in response to salinity has been attributed to the salinity effect on both stomatal and non-stomatal controls [70]. However, the same enzyme from the halophytic wild rice, *Portesia coarctata*, decreases its catalytic activity by salt and may have intrinsic structural properties to withstand such decline [71].

Calvin cycle is mainly affected under dehydration and salinity stress in stress-sensitive plants as sunflower. However, the effect of a cold stress on this pathway is still unknown. It has been previously reported that during cold acclimation there is an increase in the availability of Pi and phosphorylated intermediates in both the pathway for Suc synthesis and the Calvin cycle, and increased capacities of enzymes in both pathways [72]. However, one consequence of these long-term changes in cytosolic Pi availability and the capacity for Suc synthesis could be to pull too much carbon out of the chloroplast via the triose-phosphate transporter. This would, in turn, reduce the capacity of the Calvin cycle to regenerate RuBP and inhibit photosynthesis. However, cold-tolerant species such as *A. thaliana *and winter cereals are able to recover their photosynthetic capacity and resume growth after several days to weeks at low temperature through cold acclimation process [72-74], being actually little evidence available about sunflower response during the days after cold stress.

There are many reports on defense signals induced by different stresses including cold, salinity and drought [8,35,75-77]. In this category we report ten genes with differential expression patterns (see Additional file 1). One of them is up-regulated under both stresses (EST T411, similar to a plastidic aldolase) [GenBank: BU671801], a second one is down-regulated under chilling stress (EST T340, similar to a chloroplastic glutamine synthetase) [GenBank: BU671843] whereas a third one is specifically down-regulated under salt stress conditions. Plastidic aldolase genes characterized in *Nicotiana *plants can be grouped in two sub-families: AldP1 and AldP2. It was first reported that AldP2 was up-regulated by salt stress, whereas AldP1 was suppressed by salt stress [78]. Thus, EST T411 identified in this work as up-regulated in both stresses would be hypothetically similar to an AldP2 type due to the up-regulation observed under salinity stress. On the other hand, down regulation of the transcriptional profile of EST H136 (similar to a chloroplast drought-induced stress protein) [GenBank: O04002] under chilling and salinity was observed. Typically, pathways leading to CO_2 _fixation and light harvesting are suppressed under abiotic stresses; although there is evidence of an over expression of glutamine synthase to enhance salinity tolerance in plants [79].

#### Signaling and transcription machinery

Regulatory proteins as TFs (bZIP, MYC, MYB and DREB) as well as protein kinases and proteases are involved in transcriptional changes under abiotic stresses [5]. The activation of the transcriptional machinery in regulation of salt-dependent gene expression requires the induction of specific TFs as well as RNA polymerases [80]. Many regulatory proteins, mainly identified in *A. thaliana *[76], showed changes in TFs under environmental stresses. In this analysis, and in agreement with a previous report [35], an EST encoding a protein with similarity to a zinc finger family protein was identified as up-regulated under low temperature, although this transcription factor (TF) does not show significant similarity to the one previously reported. A zinc finger protein associated to saline stress in *Arabidopsis *was recently reported [81] which is different from the one previously identified as DREB1A [32].

Many candidate genes had been identified as TFs or as sensing receptors for calcium signaling by *in silico *analysis, showing the relative abundance of transcriptional machinery related genes in the organ-specific cDNA libraries developed by our group [50]. In the present work, two DNA binding proteins isolated by stress organ-specific cDNA libraries were detected as up-regulated specifically under salt (T187, T454) [GenBank: BU671817] [GenBank: BU671860] and a zinc finger protein specifically induced under chilling treatment. The large amount of TFs identified in *Arabidopsis*, indicating the complexity in the secondary metabolism of the plants [82], could explain the dramatic implication of those proteins in the abiotic stress responses beyond the critical interaction between plants and the environment and the level of duplications found in the *Arabidopsis *genome [83]. *A. thaliana *TFs involved in stress response are traditional classified in ABA-dependent and ABA-independent regulatory pathways. According to microarray analysis in this species there are several independently responses to abiotic stress, one of them involving the DREB/CBF regulon [34]. While DREB1 genes are specifically induced by cold, DREB2 genes are induced by dehydration and high salt but not by cold [7,32,84,85]. This response was also reported in rice [33].

Recent studies have reported the importance of HD-ZIP TFs in response to drought in an independent pathway respect to the DREB transcriptional cascade [86]. In *Arabidopsis*, ESTs libraries analysed by digital northern represented a 13% of signaling associated genes [87]. The up-regulation of an ADP-ribosylation factor described in this work (EF127) [GenBank: BU671885] would explain the regulation of the intracellular traffic through vesicles [88]. In addition, another gene under-regulated in both stresses (T234) [GenBank: BU671830] highly similar to an extracellular Ca^2+ ^sensor was detected. That receptor was recently identified in *A. thaliana *[89]. The authors demonstrated that Ca^2+ ^extracellular level regulates Ca^2+^-dependent intracellular signaling through specific sensors. In this way those receptors would modulate calcium-dependant kinases previously described as enzymes highly expressed under chilling acclimation mainly. This knowledge was reported for *Medicago sativa *plants evaluated after 10 minutes exposition at low temperatures [90,7]. The role of calcium-dependent protein-kinases under different environmental stresses was also reported in *A. thaliana *[91] and rice [92].

#### Translation machinery

In general, genes involved in this cellular process were up-regulated under abiotic stress as a protective mechanism against key enzymes activity (see Additional file 1). Regulation of the translational machinery is considered an integral component in the cellular stress response [77,93]. It has been indicated that ribosomal proteins are not only central to translational efficiency but have extra-ribosomal functions [94]. In the present assay, this is confirmed by up-regulation of ribosomal proteins under both stresses. Besides, cDNAs encoding elongation factors were detected as salt induced, as previously reported for stress-associated genes in several systems [8,95].

#### Protein turnover/folding/protein interactions

Protein degradation during stress is a highly conserved and regulated phenomenon in all the organisms reported so far [96]. In this analysis, EST F443 [GenBank: BU671999] similar to a copper chaperone from tomato, was down-regulated under both stresses, as previously reported by a MALDI-TOF analysis of cold stress induction in rice seedlings [97]. This tomato's chaperone seems to play a role in copper mobilization from decaying organs towards reproductive structures, contributing to growth in other parts of the plant [98]. In addition, EST F231 [GenBank: BU671928] similar to a cyclophilin, was also down-regulated under chilling and salinity. Three peptidylprolyl isomerases (PPI) were detected in *Arabidopsis *plants treated with NaCl being one of them similar to a cyclophilin down-regulated under salinity stress [61].

By contrast, EST T124 [GenBank: BU671806] similar to a heat shock protein was up-regulated in both stresses. Heat shock proteins (HSPs), often called the stress proteins, are now recognized as important to a range of physiological and cellular functions under both normal growth conditions and in response to stresses other than heat shock [99]. Starting in the mid-1980s, the concept of molecular chaperones evolved from the work of biochemists and cell biologists, and several HSPs were soon recognized as having such chaperoning functions. Proteomic analysis allowed the identification of several HSP's up-regulated in poplar under chilling stress, being one of them strongly similar to one of *H. annuus *detected in this work [38].

#### ROS-scavenging network

Cold stress, salinity and drought, combined with high light conditions, result in enhanced production of ROS by the photosynthetic apparatus because these conditions limit the availability of CO_2 _for the dark reaction, leaving oxygen as one of the main reductive products of photosynthesis [100]. Drought, salt, and cold stress all induce the accumulation of ROS such as super oxide, hydrogen peroxide, and hydroxyl radicals [75]. H_2_O_2 _is generated in peroxisomes by the enzymatic activity of glycolate oxidase [42]. In this study an EST with homology to a glycolate oxidase (T120) [GenBank: BU671805] was up-regulated under both stresses, probably involved in a general generation of ROS under different abiotic stresses. These ROS may be signals inducing ROS scavengers and other protective mechanisms, as well as damaging agents contributing to stress injury in plants [101]. Many ESTs from leaf and stem cDNA libraries encoding peroxidases, thioredoxins, catalases and oxygen-evolving enhancer proteins showed transcriptional changes in response to the studied stresses. Most of those proteins were up-regulated in both stresses due to the accumulation of these products along the oxidative stress. However, a NADH-plastoquinone reductase and a catalytic hydrolase were down-regulated. Genes encoding proteins associated with cellular homeostasis (respiration, cellular biogenesis and DNA repair) showed a distinct decline under abiotic stresses [37].

#### Transport

Ion homeostasis during salt stress is affected by sodium fluxes, transport and compartmentalization [8]. Abundant transport-related genes have been described in a differential gene expression study that involve hybrid sunflower species, as preferentially expressed in *Helianthus deserticola*, a xerophytic species restricted to desert habitat [102]. These genes seem to be important in the extreme environment of desert soil, functioning as both osmotic sensors and ionic regulators to prevent desiccation [103,104].

In this work, EST H360 [GenBank: BU672086] with similarity to an ATP synthase was up-regulated in both stresses, as happens with EST F557 [GenBank: BU672042] similar to a putative carrier protein. These transcriptional changes could take place as a result of the large activity of ion transporters during salt tolerance and potassium nutrition [105]. Potassium transporters may function in the transport of K^+^, which is an essential cofactor for many enzymes [75]; or control K^+ ^uptake and regulate Na^2+ ^uptake, which can be an important determinant of salinity tolerance [12,106]. Moreover, carrier proteins, water-channel proteins and sugar transporters are thought to function through plasma membranes and tonoplast to adjust the osmotic pressure under stress conditions [21]. On the other hand, lipid transfer proteins (LTPs) are another group of transport-related proteins associated to fatty-acid metabolism which may have a function in repairing stress-induced damage in membranes or changes in the lipid composition of the membranes, perhaps to regulate permeability to toxic ions and the fluidity of the membrane [107,108]. Many LTPs have been shown to affect cell wall extensibility or to be secreted in response to NaCl stress [109]. Nearly half of the detectable LTP transcripts in *Arabidopsis *root microarray were down-regulated by NaCl treatment [61]. Moreover, LTPs with similarity to *Arabidopsis*' LTPs were also detected as down-regulated in sunflower under chilling stress [35]. In the present work we identified an EST (EF502) highly similar to an LTP protein [GenBank: BU671910] being up-regulated under chilling stress and down-regulated under saline environment.

#### Secondary metabolism

EST H123 [GenBank: BU672069], which shows a high identity to a myo-inositol phosphate synthase (MIPS protein, isomerase involved in inositol metabolism) [IUBMB enzyme nomenclature: EC 5.5.1.4.] was down-regulated in chilling and salinity. Inositol is a natural cell wall osmoprotector subcellular synthesized into phosphatidyl-inositol as part of a complex process and then recycled into phosphatidyl-inositol cycle as a complex signaling mechanism under abiotic stress conditions. Transgenic tobacco tolerant to salinity mediated by a MIPS gene product has been also described in *P. coarctata *[110]. By contrast, in sesame, down-regulation by salt stress in seeds during germination was reported for the SeMIPS1 gene [111]. In addition, transcription of the MIPS gene was found to be affected by salinity during biosynthesis of myo-inositol and its derivatives [112,113], whereas evidence was reported that expression of the MIPS gene is up-regulated during salinity stress in salt-tolerant plants, while its transcriptional levels are reduced in salt-sensitive *A. thaliana *[114]. The down-regulation of EST H123 [GenBank: BU672069] in sunflower (salt-sensitive crop) reported here is strongly similar to a MIPS gene product in sesame [GO Term GO: 0004512] [50].

## Conclusion

This work presents the first cDNA sunflower fluorescence microarray analysed by a combined statistical method, studying transcriptional changes in early responses to chilling and salinity. The statistical approach to select candidate genes combining a classical hypothesis test of equal mean across treatment, i.e ANOVA, and an ordination technique based on principal component analysis appears as an efficient methodology to identify differentially expressed genes revealing a total of 80 candidate genes either under chilling and/or salt stress. Even when this represents a high percentage (28%) of differentially expressed genes from the initial number of organ-specific sequences evaluated, this is a lower proportion than that identified by the ANOVA which was about 50%. The reduction on the number of proposed differentially expressed genes by the combined selection criteria mentioned above is useful to prevent against false discoveries. Ten candidate genes from 12 selected ESTs representing different expression patterns were successfully validated by Real-Time quantitative PCR. Out of the 80 candidate genes, 50 genes were found up or down-regulated under abiotic stresses, supporting common regulatory mechanisms and general responses to low temperature and salinity. In addition, 15 and 12 genes were up or down-regulated respectively under one specific stress whereas the three remaining genes showed a contrasting transcriptional profile, being induced under one stress and suppressed under the other. Interestingly, almost half of the differentially expressed genes (39) detected in this study correspond to genes with unknown predicted function. This result indicates that even though ESTs database for *Compositae *plants comprises a large number of sequences (509,904), many of them do not have an assigned putative function. The difficulty in finding orthologous pairs in sequence comparisons with a fully sequenced genome species as *Arabidopsis *which is highly divergent from the *Compositae *species, could explain the large number of unknown or unclassified ESTs in expression studies involving partially sequenced genomes as sunflower.

There are many efforts from different research groups reporting the use of ESTs in microarrays analysis to contribute to the identification of those candidate genes whose expression level changed in presence of abiotic stresses [21,35,37,61,62,115]. However, many EST collections are not complete and are derived from cDNA libraries of plants grown under non-stressed conditions, being defense/stress ESTs under represented. In the case of sunflower, functional genomic studies targeting different forms of water-deficit stress [49,89] have been conducted using low scale thematic microarrays, though there are an important number of ESTs available for this species. In this sense, we consider that this work makes an essential contribution to the knowledge of an oil crop plant genome by its transcriptome characterization under two abiotic stresses: chilling and salinity. It represents the first work studying concerted gene expression of sunflower in response to salinity, allowing the identification of genes involved in common regulatory mechanisms to both stresses from those specifically associated to either chilling or salinity. Further studies exploring profile expression of these candidate genes under different low temperatures and salinity treatments combining different stress intensities and different stress extension periods will help to understand their role at different points of the complex regulatory mechanisms associated to stress response. Selected candidate genes could be used ultimate to test their molecular functions using expression studies for over expression or suppression of target genes in transgenic plants. These candidate sequences constitute at the same time a valuable tool to develop functional molecular markers based on SNPS/IndDels to assist selection in breeding programs.

## Methods

### Plant material

Sunflower (*H. annuus L*.) plants belonging to inbred line HA89 provided by sunflower breeding program from EEA INTA Balcarce, Argentina were grown under standard conditions in greenhouse (16 h photoperiod and 20–24°C temperature) in pots of 1 liter (volume) containing composite soil. Three pots, representing three biological samples, each of them containing 4 seedlings, were grown for each treatment including control plants, watered daily with tap water and fertilized weekly with Hakaphos (COMPO^®^) 18-18-18 (NPK) at a final concentration of 100 ppm (0.55 W/V) during 2 weeks.

### Chilling and salinity treatments

For high salinity treatment, 2-week old seedlings (2-full expanded leaves) were watered with 150 mM salt solution for three days (adapted from Liu and Baird) [48]. Control seedlings were watered with tap water and grown under the same conditions. For chilling stress, sunflower seedlings at the two-leaf stage were subjected to 10°C (adapted from Huang *et al*.) [46]. All plants were grown in growth chamber (Conviron^®^) during 24 hour with a 16 photoperiod provided by daylight fluorescent tubes (Philips, Argentina). Leaves from control and stressed seedlings were collected separately per biological replicate, immediately frozen in liquid nitrogen and stored at -80°C until processed for RNA extraction. Three biological samples represented by three pots, each one composed by four seedlings, were processed and evaluated independently for each stress treatment: chilling, salinity and control.

### Amplification and preparation of cDNAs for microarrays construction

Sunflower EST clones derived from different organ-specific cDNA libraries were grouped in contigs using BioPipeline [50]. Running BLASTN and BLASTX routines [116], 319 sunflower sequences with significant similarities (E value < 1.0 E^-10 ^or BLAST score > 80) to already known or predicted genes involved in main stress/defense responses, primary metabolic pathways, gene expression or signal transduction were selected for microarray construction. BLAST-based GO term prediction application were used to assign functional categories to the defined unigene collection considered all three GO categories at the most specific term as described in a previous work [50]. GO annotation updates were done through AmiGO [65].

### EST libraries amplification

Recombinant plasmid preparations from different organ-specific cDNA libraries [50] that were previously used for 3' sequencing were used again for PCR amplification. LacZ1 and 2 forward and reverse primers were used to amplify the cloned inserts in 96 well plates using an Eppendorf thermocycler.

(LacZ1) 5'-3' sequence: GCT TCC GGC TCG TAT GTT GTG TG 5'-3'

(LacZ2) 5'-3'sequence: AAA GGG GGA TGT GCT GCA AGG CG.

Four PCR reaction plates were prepared, one from each cryogenic glycerol tube containing the cloned EST. Long Expand Template PCR System kit (Roche Diagnostics, Inc.) was used in a 50 μl final volume master mix consisting of final concentrations of 1× PCR buffer 3, dNTPs 0.2 mM, 0.25 mM Lac Z primers, Taq Pol Mix 0.75 u. Forty eight μl from this volume were aliquoted into each well of a 96 well PCR reaction plate (MJ Research). A 2 μl aliquot of an undiluted plasmid template DNA was aliquoted into the 48 μl of master mix. The plates were briefly centrifuged for 30 sec at 1500 rpm and placed into a Eppendorf thermocycler at denaturing conditions (94°C) for 1 min, followed by 32 cycles of 94°C for 30 sec; annealing temperatures were programmed at a descendant ramp from 60°C to 55°C and 72°C for 30 sec and a final extension of 72°C for 5 minutes. A typical yield from the PCR was about 50–150 ng/μl of amplified DNA.

### Purification

The PCR products were loaded into a 96-well plate (Millipore #MANU3050) and vacuum filtered at 15 psi for about 10 min until the wells were completely empty. Purified products (QIAquick 96 PCR Purification Kit, Qiagen, Germany) were eluted in sterile water. One aliquot from each sample was evaluated by electrophoresis on 1% agarose electrophoresis gels to confirm amplification quality and quantity. Low DNA Mass Ladder (Invitrogen 10068013, Invitrogen, Argentina) was simultaneously run in the same electrophoresis gels to get a reliable PCR product quantification by fluorescence comparison using a Typhoon (GE Healthcare Life Sciences, Argentina) digitalization machine and software.

### Spotting, microarray construction and post-print processing

Five μl form each of the purified PCR cDNA products (317 in total) were transferred into a 384 well plate containing 5 μl of DMSO 100% and spotted onto coated glass-slides (Ultragap II, Corning Systems, USA) by Gentron Genomic Services (Gentron, Buenos Aires, Argentina), using VersArray Chip Writer (BioRad, USA). The array design consisted in 4 supergrids, each containing 6 subgrids of 64 spots each (8 × 8), being each cDNA printed in quadruplicate. Three clones corresponding to house mouse (*Mus musculus*) were used as negative controls [GenBank: NM009060, NM008690, NM019476] while actin and rRNA sequences from sunflower (*H. annuus*) [GenBank: AAF82805] were used as a positive control for expression analysis in all microarray slides. The printed arrays were cross-linked to the slide by UV irradiation at 250 mJ using UV Stratalinker 2400 (Stratagene, USA). The slides were stored in a dessicator chamber until use. All the slides were hybridized with a pooled control RNA used as reference hybridization.

The microarray derived data platform was entered in The Gene Expression Omnibus database [117,118] from which a platform accession number was assigned [GEO: GPL 4366]. Thus, complete tables of sequence identifiers and organ-specific unigenes accession numbers printed on arrays are available [50].

### RNA, extraction, purification, amplification and labeling

Total RNA was extracted from approximately 2 g of leaf tissue using TRIzol^® ^reagent following manufacturer recommendations (Invitrogen, Argentina). RNA integrity was analyzed by checking its electrophoretic mobility on 1.5% agarose gels in ME buffer (400 mM MOPS, 100 mM Na acetate, 10 mM EDTA pH 8.0, in diethyl-pyrocarbonate treated water). RNA was further purified by use of RNeasy Mini columns (Qiagen, Germany) according to manufacturer's instructions. To control biological variation between individuals, three biological samples from the same tissue were pooled on one sample prior to probe preparation. The reference (control) sample consisted of pooled RNA extracted from sunflower seedlings growing under unaltered environmental greenhouse conditions, whereas chilling and salinity samples were RNA extracted from sunflower seedlings growing in greenhouse under those stressed conditions.

The RNA (800 ng) samples were labeled by using SuperScript Indirect RNA Amplification System Kit (Invitrogen, Argentina) based on the method previously reported [119]. Following RNA amplification (with the incorporation of UTP aminoallyl), labeled product was achieved by incubating with Cy3 or Cy5 esters in alkaline media.

### Microarray hybridization reactions

The microarray slides were used in order to quantify the relative expression of ESTs in control and treated leaves by Cy3 and Cy5 hybridization technique. Dye-swaps were used to correct for differences in incorporation and fluorescent properties of both dyes, generating a number of 9 slides per experiment (three slides for control and three slides for each treatment) with a total number of 18 slides considering dye-swaps hybridizations. The microarray slides were prehybridized by incubation in 5× SSC, 0.1% SDS, 1% BSA at 42°C. In the next day, the cover slip was removed and the slide was washed once in 1× SSC, 0.2% SDS (prewarmed to 42°C); once in 0.2× SSC, 0.2% SDS at room temperature; and once in 0.1× SSC at room temperature. Washes were conduced with gentle shaking at 100 rpm for 5 minutes. Slides were subjected to low speed centrifugation for 2 min at 500 rpm to dry them.

### Slide scanning and signal quantitation

The hybridized slides were scanned using a VersArray Chip Reader (BioRad, USA) scanner (two different channels for the two different dyes were used) at three different detector sensitivities. Image analysis and signal quantification were performed using free open source software Spotfinder [120], quantifying signal intensity for each spot. Then, data integration from multiple scanning processes was achieved.

### Data normalization – Normalization within microarrays

Background subtraction was performed before calculating ratios. The elements with either printing or hybridization artifacts were flagged and discarded before analysis. Only spots with an intensity of at least 1.5 times above the local background in both channels were used for subsequent analysis. The outcoming data from each slide were then log transformed (using log base 2) and normalized using 3-D normalization (depending on spot intensity and it's location in the array) (Alvarez *et al*., unpublished data) using "The R statistical language" [121]. Potential artifacts and false positives were eliminated and only those clones that exhibited similar expression patterns between the original hybridization and the corresponding dye swaps were selected for further analysis [122]. A gene expression matrix was generated and its analysis was focused on differentially expressed genes.

### Normalization between microarrays

Methodology used among biological replicates hypothesize that most of genes do not change their expression level among treatments. In this context, quantiles equalization or other normalization tools will not substantially modify the change to detect patterns of different expression levels. However, according to exploratory data analysis it was determined that an important fraction of the organ-specific ESTs did show some expression level differences among treatments. So, no additional normalization was applied under the risk of increasing the false positive or negative identification discovery rates. In order to have gene profiles ranged according to their experimental error, the gene expression matrix was scaled in a gene by gene basis, dividing by the common within-treatments standard deviation, thus generating the Gene Expression Matrix (as it is referred in this work).

### Gene expression matrix analysis

The whole analysis related to gene expression matrix was performed using software Infostat 2006^® ^[123]. A two step procedure was used for candidate gene identification. First an analysis of variance for every gene was performed and only those genes having p-values lower that 5% were retained for complementary analysis. The analysis of variance was run for a fixed effect model under a complete random design. In a second step, the location of genes in the space spanned by the two first principal components of the gene expression matrix was used to filter genes and reduce the rate of positive false discoveries. The rationality behind this procedure is that the farther the gene is to the origin of the space, the larger the fold change expression level. Under this assumption, those genes located in the periphery of the resulting bi-plot should be the most dramatically involved in the responses to stress. The cut-off criterion was set as the distance-to-the-origin of the EST T411 (contig of the EST T111, AN: BU671801). The main basis of using this EST as cutting point was that it had been already experimentally validated as differentially expressed by Northern-blot and qRT-PCR. The position of EST T411 in the distance-to-the-origin distribution corresponds to the 70th percentile.

A graphical representation of the gene expressions among treatment conditions is presented as a heatmap plot (see Additional file 2). The average linkage method with Euclidean distance was used to generate the clustering relationships using the *heatmap *function in "The R statistical language" [, #241].

### Northern blotting

For northern blot analyses total RNA (20 ug) from leaves was fractionated on 1.5% agarosa-MOPS 1× gel and blotted onto nylon membranes (Hybond-N+, GE Healthcare Life Sciences, Amersham, Argentina). In all cases, membranes were cross-linked by UV illumination. Probes used for northern hybridization were prepared by random priming of the purified PCR products corresponding to EST candidate gene clone using ^[32P]^-dCTP (NEN Perkin Elmer, USA). Hybridizations were performed at 42°C in the presence of 50% formamide (Ambion ULTRAhyb^® ^Ultrasensitive Hybridization Buffer, Ambion, USA) and washes were also done at 42°C, according to provider's instructions. Exposed to BIOMAX MR Kodak X-ray films (KODAK, SIGMA Argentina) were scanned and analyzed with the TN-image program in a Typhoon device to calculate the relative signal intensities standardized with respect to rRNA and actin sequence from sunflower [GenBank: AAF82805] signals.

### Real-time RT-PCR

To confirm the results obtained from microarrays experiments, the transcript abundances of 10 differentially expressed ESTs were tested. Gene-specific primers were designed using Primer 3 [124]. Oligonucleotide primer sequences are shown in Table 1. First-strand cDNA was reverse transcribed from 500 ng of DNase treated RNA according to manufacturer instructions (Invitrogen, Argentina). The reaction was performed in a 30-ul volume containing 15 ul QuantiTect™ SYBR^® ^Green PCR (Qiagen, Germany), 300 nm of each primer and 1 μl of cDNA derived from RT product. The PCR reactions were run in an ABI PRISM 7000 HT Sequence Detection System (Applied Biosystems, USA) using the following program: 50°C for 2 min, 95°C for 10 min and 40 cycles of 95°C for 15 sec and 60°C for 1 min. Following PCR amplification, the reactions were subjected to a temperature ramp to create the dissociation curve, measured as changes in fluorescence readings as a function of temperature, allowing the detection of non-specific products. The dissociation program was 95°C for 15 sec, 60°C for 15 sec, followed by 20 min of slow ramp from 60°C to 95°C. Three replicates of each reaction were performed and actin sequence from sunflower [GenBank: AAF82805] was used as an internal control to normalize gene expression level. Negative control reactions using untranscribed RNA were also run to confirm absence of genomic DNA. Quantifying the relative changes in gene expression was performed using the 2^-ΔΔCT ^method [51]. Comparative results between qRT-PCR and microarray fold changes are presented in Table 2.

## Authors' contributions

PF carried out subtracted cDNA libraries, DNA sequencing, DNA amplification for cDNA spotting, hybridization probes and participated in data analysis and manuscript preparation; JDR directed data microarray and statistical analysis, LF participated in quantitative real time reactions, HEH coordinated the workgroup, NP directed bioinformatics and analytical routines and RH designed the experiment, coordinated the whole analysis and drafted the manuscript. All authors read and approved the final manuscript.

## Supplementary Material

Additional file 1 **Candidate genes (80) detected among treatments according top-value in median comparison and PCA analysis**. (1) Functional category is based on the Gene Ontology Project. Letters in brackets refer to the corresponding ontology category (B: biological process, C: cellular component and M: molecular function). (2) The "relative to control expression" column indicates gene expression change as "Up"/"Down" regulated or no changed ("NC") compared to the control, based on the analysis of variance. Differences between control and stress conditions are evaluated considering the variance of experimental error of each gene. Click here for file

Additional file 2 **Expression profile of selected candidate genes**. Ctrl1 = control leaf 1, Ctrl2 = control leaf 2, C1 = cold leaf 1, C2 = cold leaf 2, C3 = cold leaf 3, S1: salinity leaf 1, S2 = salinity leaf 2, S3 = salinity leaf 3. Click here for file

Additional file 3 **Heatmap plot of the 80 genes identified as differentially expressed between treatments**. Ctrl1 = control leaf 1, Ctrl2 = control leaf 2, C1 = cold leaf 1, C2 = cold leaf 2, C3 = cold leaf 3, S1: salinity leaf 1, S2 = salinity leaf 2, S3 = salinity leaf. 3. Click here for file

Additional file 4 **qRT-PCR for differentially expressed candidate genes**. Normalized report (ΔRn) vs. cycle for the ten candidate genes validated. CtrlL = control leaf, CL = cold leaf, SL: salinity leaf. Sunflower actin [GenBank: AAF82805) was used as reference "housekeeping" gene. Three biological samples were tested starting from the same RNA used as microarray hybridization probe. The average value for each of them was calculated and analysed in the graph. Click here for file
