# Supplementary material for: Transcriptomic identification of candidate genes involved in sunflower responses to chilling and salt stresses based on cDNA microarray analysis
Source: BMC Plant Biol. 2008 Jan 26;8:11. doi: 10.1186/1471-2229-8-11 (PMC2265713; doi:10.1186/1471-2229-8-11)
Supplement: Additional file 1 — Candidate genes (80) detected among treatments according top-value in median comparison and PCA analysis. (1) Functional category is based on the Gene Ontology Project. Letters in brackets refer to the corresponding ontology category (B: biological process, C: cellular component and M: molecular function). (2) The "relative to control expression" column indicates gene expression change as "Up"/"Down" regulated or no changed ("NC") compared to the control, based on the analysis of variance. Differences between control and stress conditions are evaluated considering the variance of experimental error of each gene. [file 1471-2229-8-11-S1.pdf]

GenBank (dbEST) Accession Number	EST name	GO classification [1]	Gene product	Accession number	Cold		Salinity	
					Relative to control expression [2]	Fold change	Relative to control expression [2]	Fold change
**Central metabolism/Photosynthesis (10)**								
BU671793	F175	GO:0009523 (C, photosystem II)	photosystem II 22-K chlorophyll-binding protein - rice	BAA12337.1	Down	-1.604	NC	-1.060
BU672070	H124	GO:0009765 (B, photosynthesis, light harvesting)	Q9SY97_ARATH PSI type III chlorophyll a/b-binding protein	AAD25555.1	Down	-1.844	Down	-1.412
BU672060	H125	GO:0009765 (B, photosynthesis, light harvesting)	(AL132962) Lhca2 protein [*Arabidopsis thaliana*]	CAB71077.1	Down	-2.246	Down	-2.015
BU672102	H136	GO:0009055 (M, electron carrier activity)	O04002_SOLTU CDSP32 protein (Chloroplast Drought-induced Stress Protein of 32kDa) general metabolisms	O04002	Down	-2.015	NC	1.186
BU672078	H302	GO:0009507 (C, chloroplast)	Magnesium-protoporphyrin IX monomethyl este	Q9M591	Down	-1.881	Down	-1.314
BU672090	H385	GO:0042132 (M, fructose-bisphosphatase activity)	fructose-1,6-bisphosphatase [*Pisum sativum*]	5305145	Down	-2.399	Down	-1.749
BU672091	H411	GO:0009538 (C, photosystem I reaction center)	SAF_FLATR PHOTOSYSTEM I REACTION CENTRE SUBUNIT III PRECURSOR (LIGHT-HARVESTING COMPLEX I 17 KD PROTEIN) (PSI-F) (*Flaveria trinerva*)	AAA33344.1	Down	-2.453	Down	-1.803
BU671800	T129	GO:0009765 (B, photosynthesis, light harvesting)	Q9SDT2_DAUCA Chlorophyll a/b-binding protein.	AAF20948.1	Up	1.262	Up	1.269
BU671843	T340	GO:0004356 (M, glutamate-ammonia ligase activity)	O04961_HELAN Chloroplastic glutamine synthetase (EC 6.3.1.2) (Fragment) general metabolism	AAN84537.1	NC	-1.046	Down	-1.522
BU671801	T411	GO:0004332 (M, fructose-bisphosphate aldolase activity)	Q9SXX5_NICPA Plastidic aldolase general metabolism	BAA77603.1	Up	1.498	Up	1.282
**Translation machinery (6)**								
BU671926	F230	GO:0003735 (M, structural	*Solanum lycopersicon* ribosomal protein L2 protein	CAA45863.1	NC	-1.071	Down	-1.180

GenBank (dbEST) Accession Number	EST name	GO classification [1]	Gene product	Accession number	Cold		Salinity	
					Relative to control expression [2]	Fold change	Relative to control expression [2]	Fold change
		constituent of ribosome)						
BU672008	F467	GO:0003735 (M, structural constituent of ribosome)	*Arabidopsis thaliana* 60S ribosomal protein L13a protein	BAB09429.1	Up	1.660	NC	1.105
BU672004	F455	No GO term	Q5I7K2_WHEAT Ribosomal protein S7	Q5I7K2	Up	1.503	Up	1.323
BU672017	F550	No GO term	Translation initiation factor B04 [*Helianthus annuus*]	21842314	Down	-1.366	Down	.1.321
BU671923	F577	GO:0003723 (M, RNA binding)	RS4_SOLTU 40S RIBOSOMAL PROTEIN S4 (potato)	AAC24585.1	Up	1.222	Up	1.306
BU671873	T111	GO:0003746 (M, translation elongation factor activity)	*Solanum lycopersicon* elongation factor 1-alpha protein	CAA37212.1	NC	-1.185	Up	1.376
**No homology (1)**								
BU672056	H110	No GO term	No homology in databases		Down	-1.618	Down	-1.208
**Transcriptional machinery (4)**								
BU671940	F305	GO:0003677 (M, DNA binding)	41 kD chloroplast nucleid DNA binding protein (CND41) [*Nicotiana sylvestris*]	24430421	Up	1.550	NC	1.171
BU672034	F561	No GO term	Zinc finger family protein (Fragment)	Q5ULY2	Up	1.547	NC	1.138
BU671817	T187	GO:0003677 (M, DNA binding)	MBD4; DNA binding [*Arabidopsis thaliana*]	15229373	NC	-1.025	Up	1.715
BU671860	T464	GO:0006118 (B, electron transport)	Single-stranded DNA binding [*Medicago truncatula*]	ABD28355.1	NC	1.225	Up	1.306
**Signaling machinery (2)**								
BU671885	EF127	GO:0003924 (M, GTPase activity)	ADP-ribosylation factor 1.	Q7F270_ORYSA	Up	1.759	Up	1.743
BU671830	T234	No GO term	Extracellular Ca sensing receptor (*Arabidosis*)	Q9FN48	Down	-2.181	Down	-1.445
**Protein turnover/folding/interactions (5)**								
BU671886	EF264	GO:0004175 (M, endopeptidase activity)	*Arabidopsis thaliana* multicatalytic endopeptidase protein	CAA73619.1	Up	1.267	NC	1.162
BU671928	F231	GO:0003755 (M, peptidyl-prolyl cis-	Q7XY71_9ROSI Cyclophilin (Fragment)	Q7XY71	Up	-1.395	NC	-1.089

GenBank (dbEST) Accession Number	EST name	GO classification [1]	Gene product	Accession number	Cold		Salinity	
					Relative to control expression [2]	Fold change	Relative to control expression [2]	Fold change
		trans isomerase activity)						
BU671999	F443	GO:0003743 (M, translation initiation factor activity)	Copper chaperone [*Lycopersicon esculentum*]	30039180	Down	-1.179	Down	-1.103
BU671806	T124	GO:0005524 (M, ATP binding activity)	Q8LKP8_SAUME Heat shock protein	Q8LKP8	Up	1.355	Up	1.520
BU671835	T289	GO:0004374 (M, glycine cleavage system)	*Flaveria pringlei* P-protein of the glycine cleavage system protein	CAA85353.1	Down	-1.395	Down	-1.305
**Transport (3)**								
BU671910	EF502	GO:0016068 (B, type I hypersensitivity)	*Gossypium hirsutum* lipid transfer protein precursor protein transport	AAF35184.1	Up	1.101	Down	-1.141
BU672086	H360	GO:0046961 (M, hydrogen ion transporting ATPase activity, rotational mechanism)	ATP synthase beta subunit [*Helianthus annuus*]	CAB65035.1	Up	1.814	Up	1.246
BU672042	F557	GO:0005488 (M, binding)	ARATH A_IG002N01.16 protein (Putative carrier protein)	AAL07192.1	Up	1.798	Up	1.368
**Secondary metabolism (3)**								
BU672069	H123	GO:0004512 (M, inositol-3-phosphate synthase activity	myo-inositol-1-phosphate synthase (EC 5.5.1.4) - common ice plant	BAA84084.1	Down	-1.429	Down	-1.515
BU672083	H322	No GO term	Q43469_HELAN Delta-8 sphingolipid desaturase	Q9ZRP7	Down	-1.288	Down	-1.467
BU671815	T322	GO:0004497 (M, monooxygenase activity)	*Glycine max* geranylgeranyl hydrogenase protein.	AAD28640.1	NC	-1.041	Up	1.548
**ROS machinery (7)**								
BU672105	H354	GO:0008137 (M, NADH	NADH-plastoquinone oxidoreductase subunit 4 [*Oenothera elata subsp. hookeri*]	CAB67220.1	Down	-1.381	Down	-1.168

GenBank (dbEST) Accession Number	EST name	GO classification [1]	Gene product	Accession number	Cold		Salinity	
					Relative to control expression [2]	Fold change	Relative to control expression [2]	Fold change
		dehydrogenase (ubiquinote) activity)						
BU671805	T120	GO:0009055 (M, electron carrier activity)	Q84LB8_ZANAE Glycolate oxidase (EC 1.1.3.15) (EC 1.1.3.1) ROS machinery	Q84LB8	Up	1.178	Up	1.292
BU671812	T155	GO:0009055 (M, electron carrier activity)	Putative thioredoxin reductase [*Hordeum vulgare subsp. vulgare*]	38174811	Up	1.467	NC	1.064
BU671825	T221	GO:0004301 (M, epoxide hydrolase activity)	catalytic/ hydrolase [*Arabidopsis thaliana*] ROS machinery	NP_849507.1	Down	-2.072	Down	-1.337
BU671832	T243	GO:0006986 (B, response to unfolded protein)	HSP7S_PEA Stromal 70 kDa heat shock-related protein, chloroplast precursor	Q02028	Up	1.509	NC	1.035
BU671838	T307	GO:0004096 (M, catalase activity)	*Helianthus annuus* catalase protein	AAA69866	Up	1.730	Up	1.801
BU671846	T368	GO:0009507 (C, chloroplast)	PSBQ2_ARATH Oxygen-evolving enhancer protein 3-2, chloroplast precursor (OEE3) (16	Q41932	Up	1.225	Up	1.306
**Unclassified**								
BU672101	EF432		No GO Term associated		Down	-1.319	Down	-1.307
BU671912	EF624		No GO Term associated		Down	-1.751	Down	-2.372
BU671986	F137		No GO Term associated		NC	-1.185	Down	-1.599
BU671987	F171		No GO Term associated		Down	-1.797	Up	1.689
BU671919	F176		No GO Term associated		Up	1.280	Up	1.163
BU671924	F192		No GO Term associated		NC	-1.085	Up	1.100
BU671945	F202		Q9SL05_ARATH Expressed protein (At2g05620/T20G20.3)	Q9SL05	NC	1.062	Up	1.669
BU671949	F209		No GO Term associated		Down	-1.263	NC	-1.044
BU671950	F210		No GO Term associated		Down	-1.773	NC	-1.165
BU671927	F216		No GO Term associated		Down	-1.941	Down	-1.938
BU671931	F295		No GO Term associated		Up	1.396	NC	1.091
BU671961	F319		No GO Term associated		Up	1.821	NC	1.029
BU671965	F340		No GO Term associated		Up	1.261	NC	1.085

GenBank (dbEST) Accession Number	EST name	GO classification [1]	Gene product	Accession number	Cold		Salinity	
					Relative to control expression [2]	Fold change	Relative to control expression [2]	Fold change
BU671980	F373		No GO Term associated		Up	1.461	NC	1.085
BU671983	F379		No GO Term associated		Up	1.306	NC	1.191
BU671988	F401		No GO Term associated		Down	-1.511	Down	-1.265
BU671788	F406		No GO Term associated		Up	2.074	Up	1.677
BU672039	F426		No GO Term associated		Up	1.387	NC	1.172
BU672013	F482		No GO Term associated		Up	1.600	Up	1.238
BU672016	F489		No GO Term associated		Up	1.469	Up	1.519
BU672017	F491		No GO Term associated		Up	1.697	Up	1.314
BU672019	F494		No GO Term associated		Up	1.272	Up	1.288
BU672020	F496		No GO Term associated		Up	1.421	Up	1.328
BU672110	F514		No GO Term associated		Up	1.389	Up	1.240
BU672026	F543		similar to unknown protein [*Arabidopsis thaliana*]	BAB02703.1	Down	-1.587	Down	-1.271
BU672028	F549		No GO Term associated		Down	-1.350	Down	-1.213
BU672032	F554		No GO Term associated		Up	1.415	NC	1.155
BU672046	F572		No GO Term associated		Up	1.344	Up	1.237
BU672051	F593		No GO Term associated		Up	1.517	NC	1.126
BU671789	H111		No GO Term associated		Down	-2.094	NC	-1.198
BU672074	H209		No GO Term associated		NC	-1.158	Down	-1.624
BU672079	H304		No GO Term associated		Down	-1.477	Down	-1.908
BU672084	H329		No GO Term associated		NC	1.110	Up	1.527
BU672087	H368		No GO Term associated		Up	1.241	Up	1.687
BU672092	H387		No GO Term associated		Down	-1.328	Down	-1.329
BU671799	T107		No GO Term associated		Down	-1.333	Up	1.348
BU671868	T253		No GO Term associated		Down	-1.593	Down	-1.645
BU671875	T283	GO:0016740 (M, transferase activity)	Os03g0651000 [*Oryza sativa (japonica cultivar-group)*]	115454377	Up	1.609	NC	1.105
BU671862	T479				Up	1.794	Up	1.470
