# Supplementary figures and images for: Transcriptomic identification of candidate genes involved in sunflower responses to chilling and salt stresses based on cDNA microarray analysis
Source: BMC Plant Biol. 2008 Jan 26;8:11. doi: 10.1186/1471-2229-8-11 (PMC2265713; doi:10.1186/1471-2229-8-11)

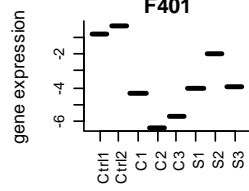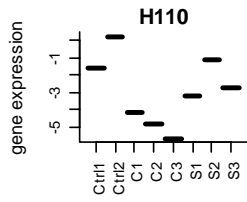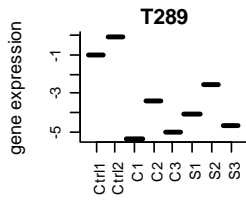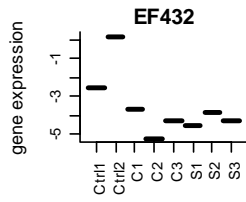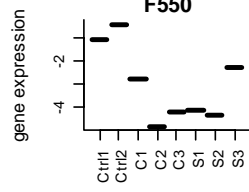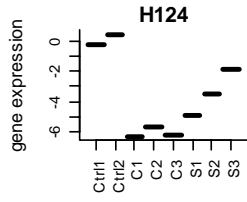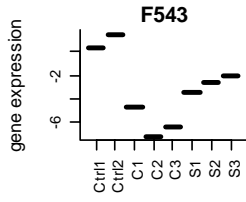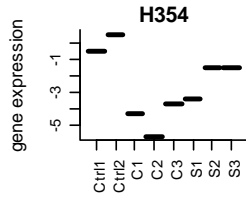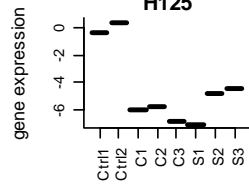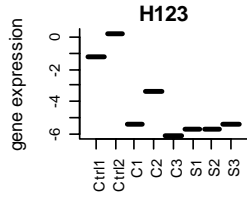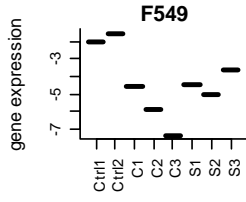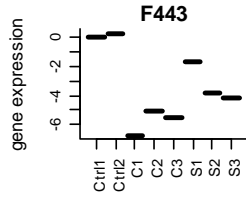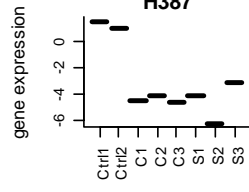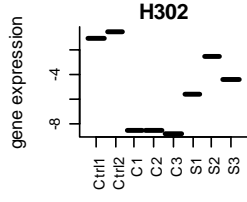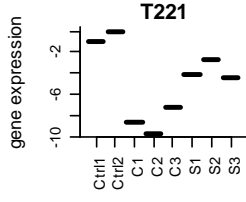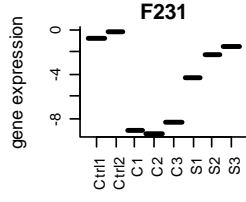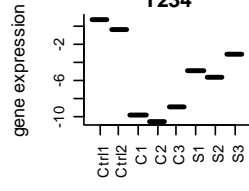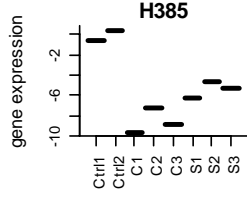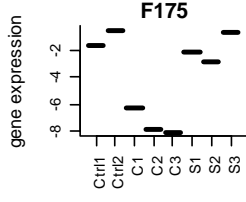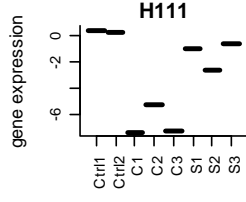

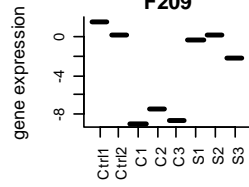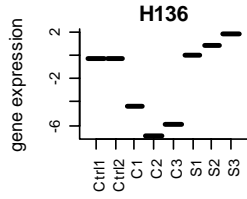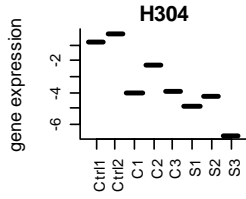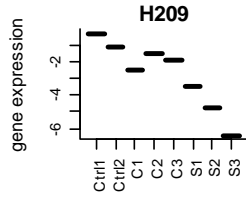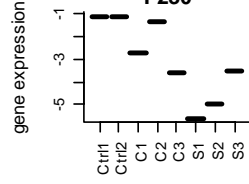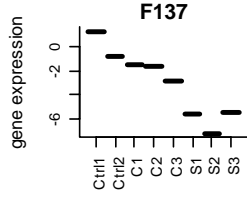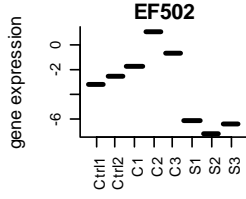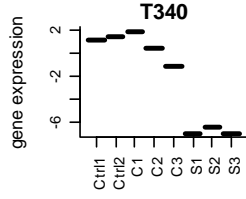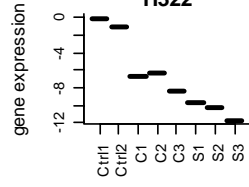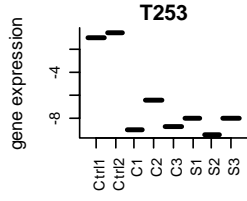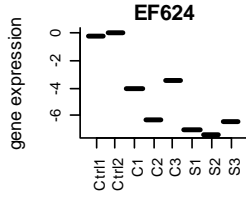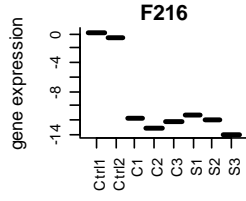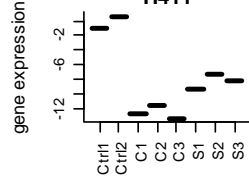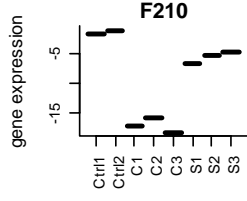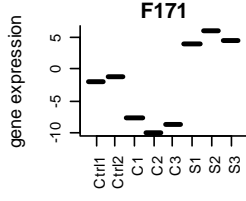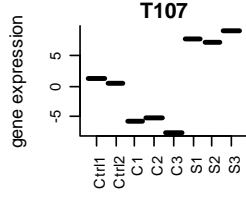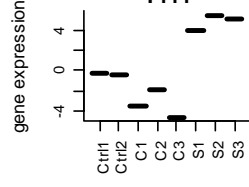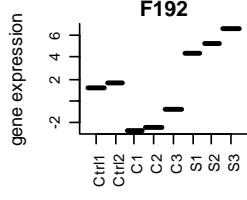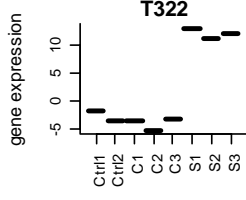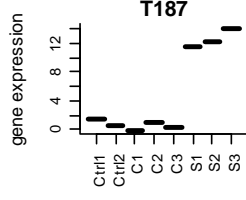

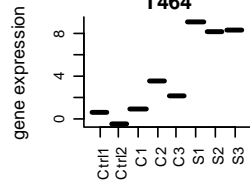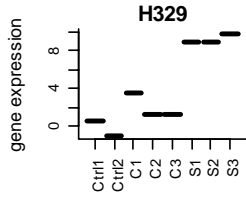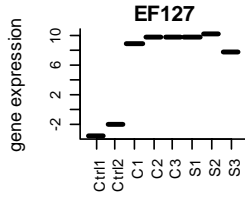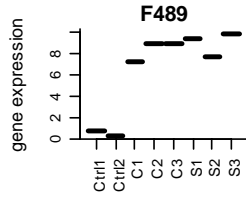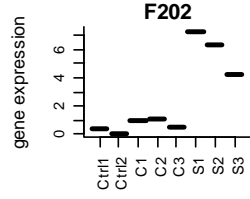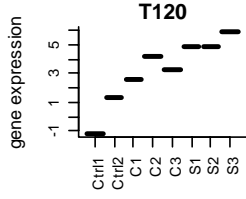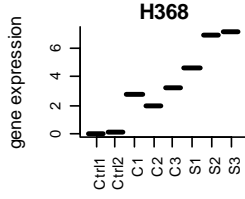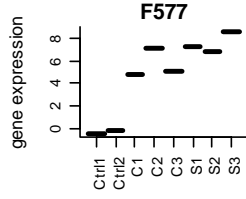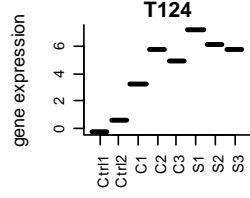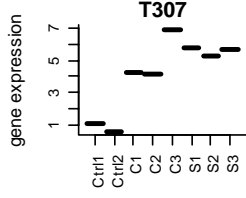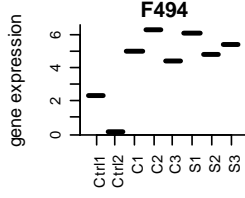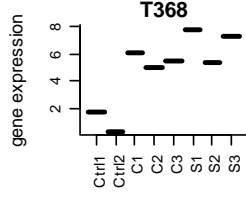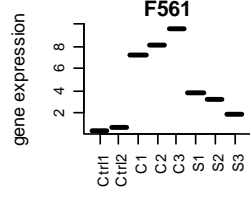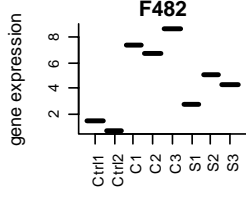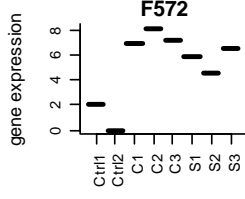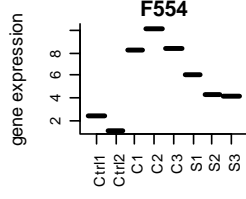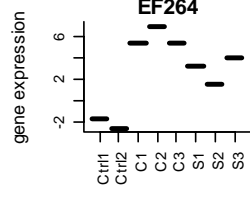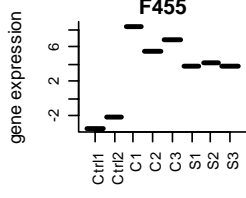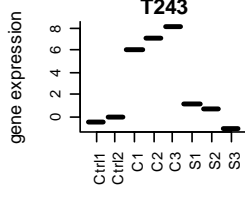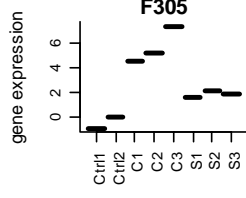

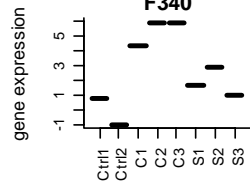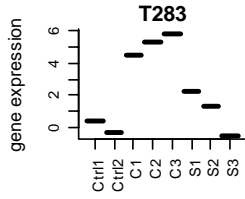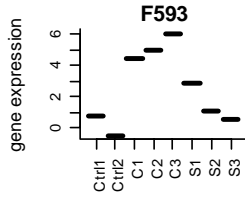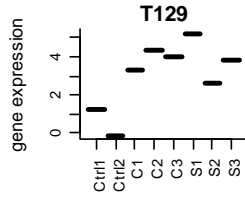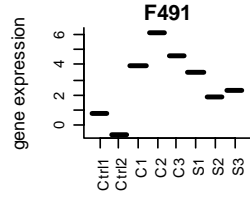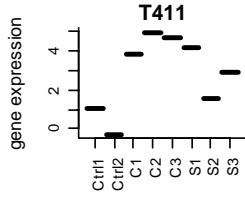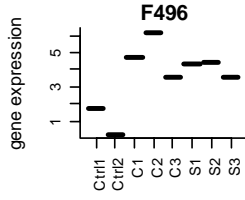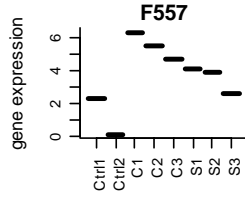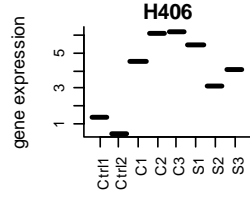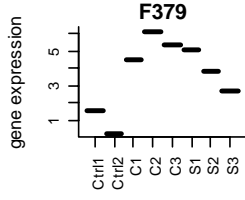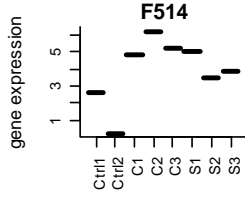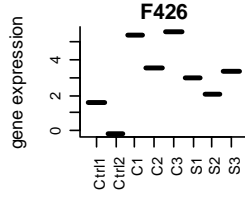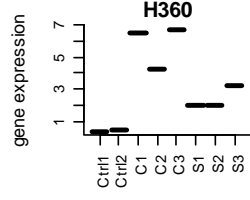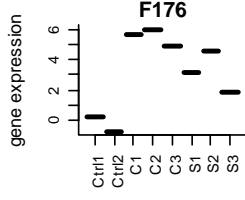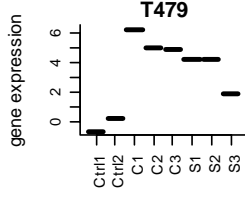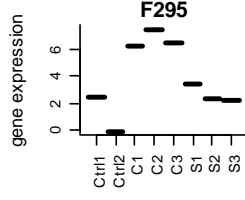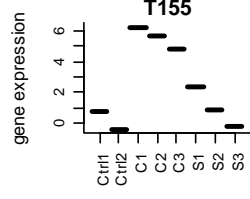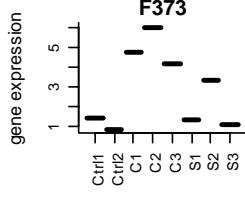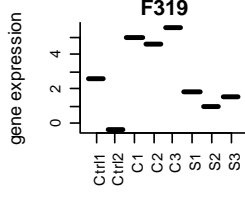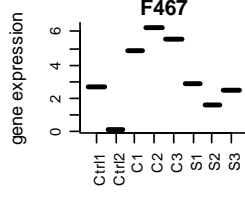

Supplement: Additional file 2 — Expression profile of selected candidate genes. Ctrl1 = control leaf 1, Ctrl2 = control leaf 2, C1 = cold leaf 1, C2 = cold leaf 2, C3 = cold leaf 3, S1: salinity leaf 1, S2 = salinity leaf 2, S3 = salinity leaf 3. [file 1471-2229-8-11-S2.pdf]

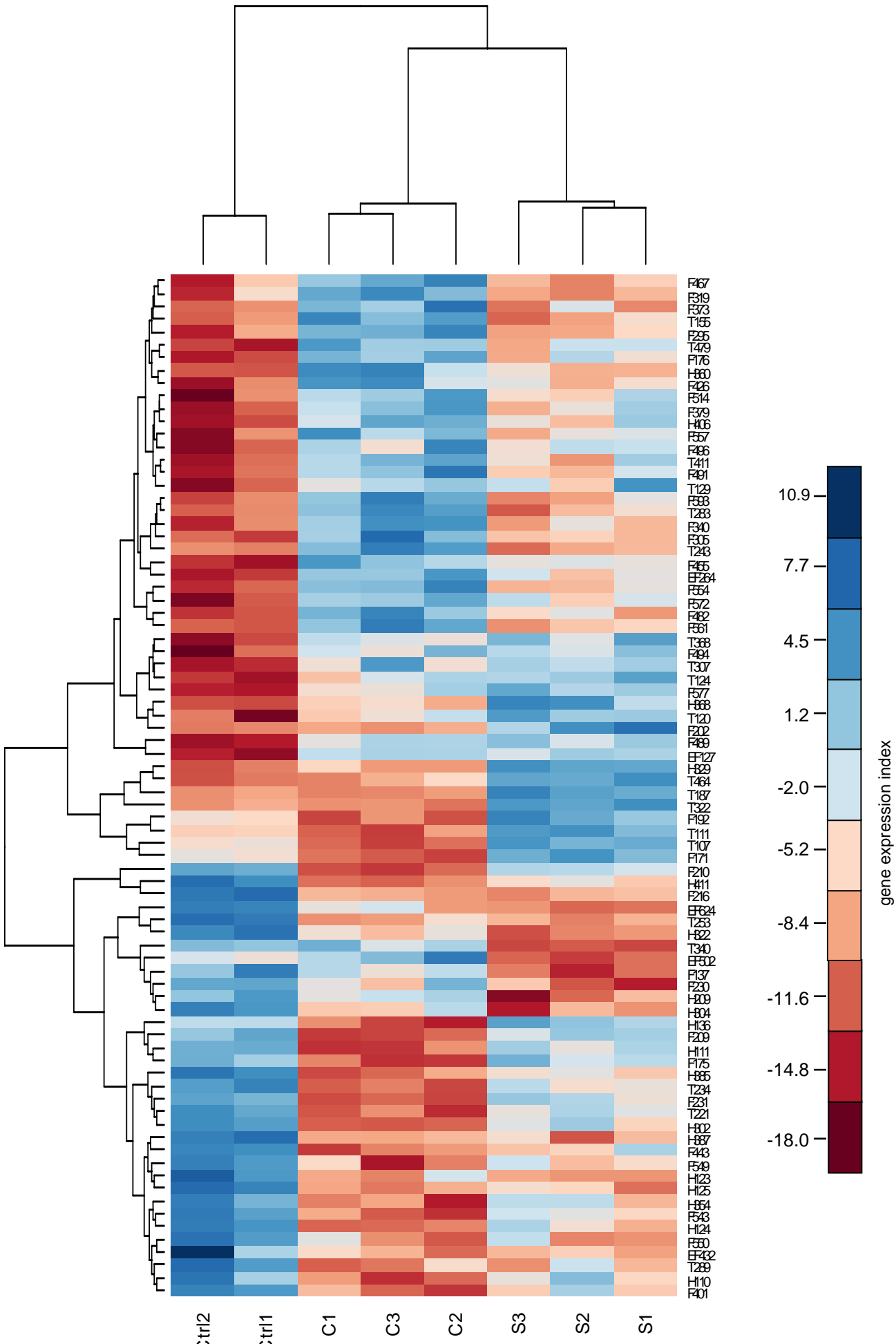

Supplement: Additional file 3 — Heatmap plot of the 80 genes identified as differentially expressed between treatments. Ctrl1 = control leaf 1, Ctrl2 = control leaf 2, C1 = cold leaf 1, C2 = cold leaf 2, C3 = cold leaf 3, S1: salinity leaf 1, S2 = salinity leaf 2, S3 = salinity leaf. 3. [file 1471-2229-8-11-S3.pdf]

EST EF127

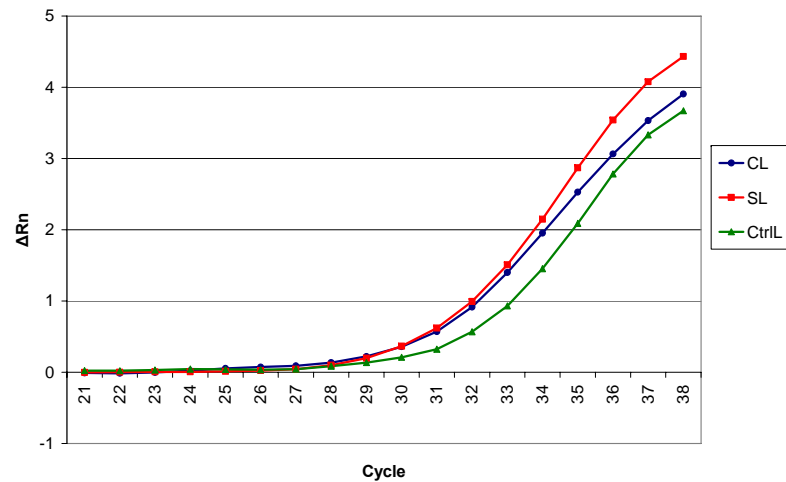

EST EF502

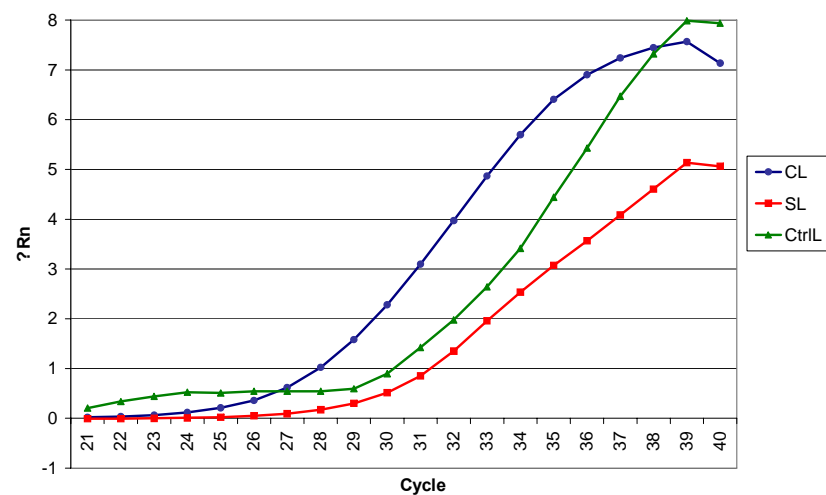

EST F171

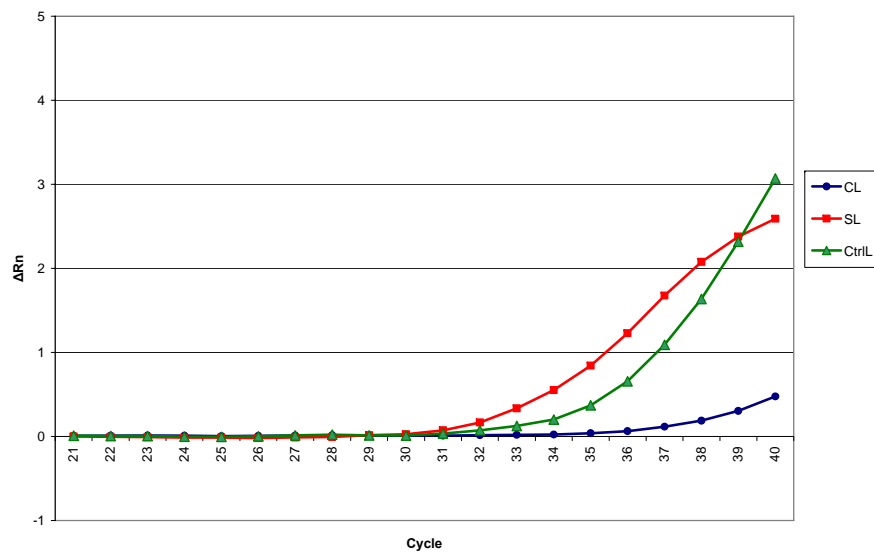

EST F455

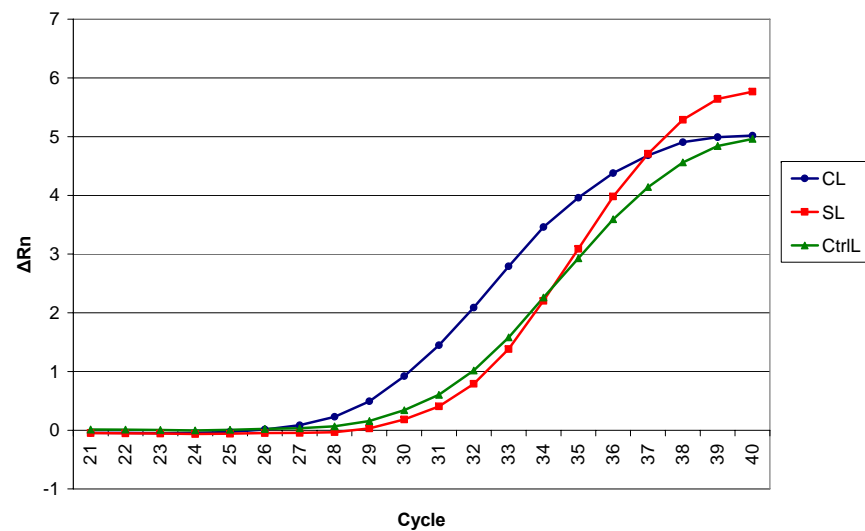

EST H360

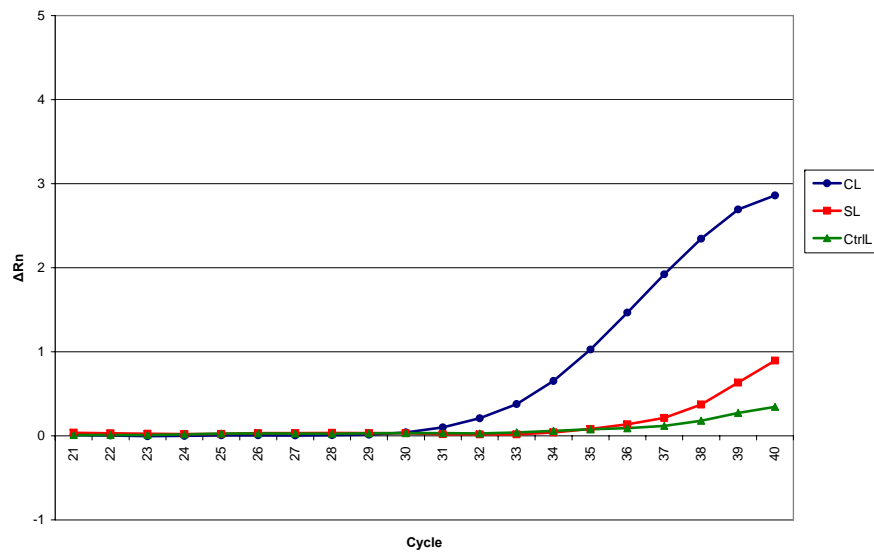

EST T124

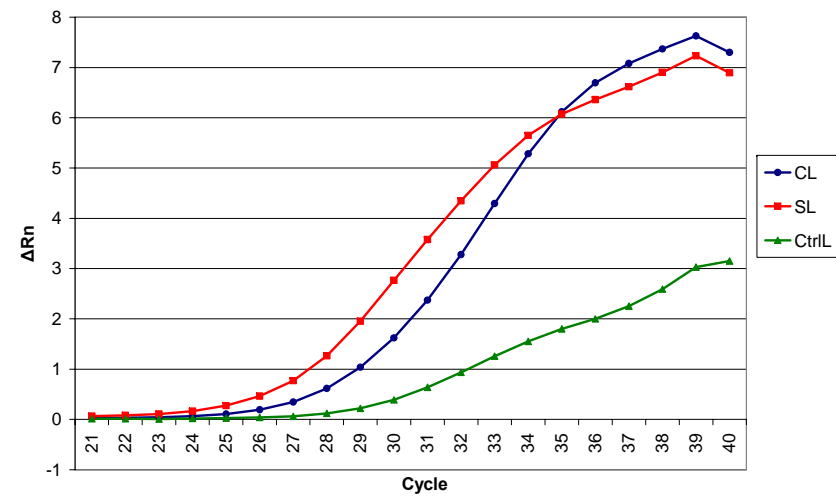

EST T411

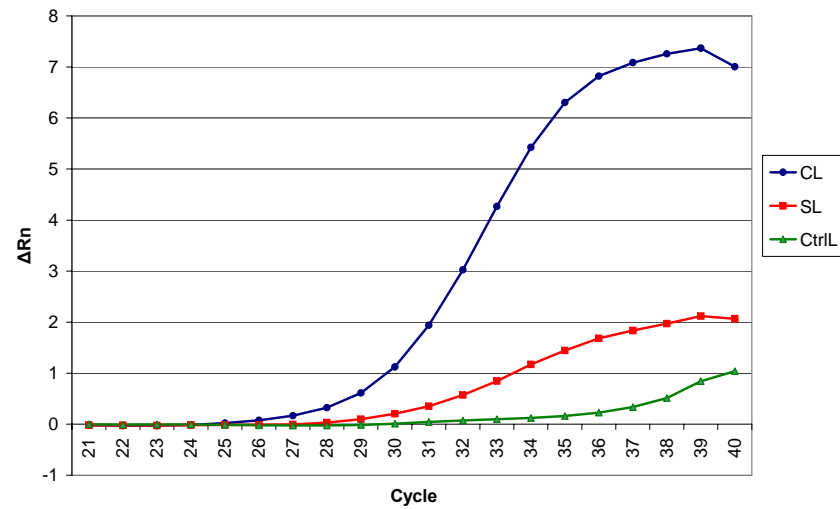

EST EF264

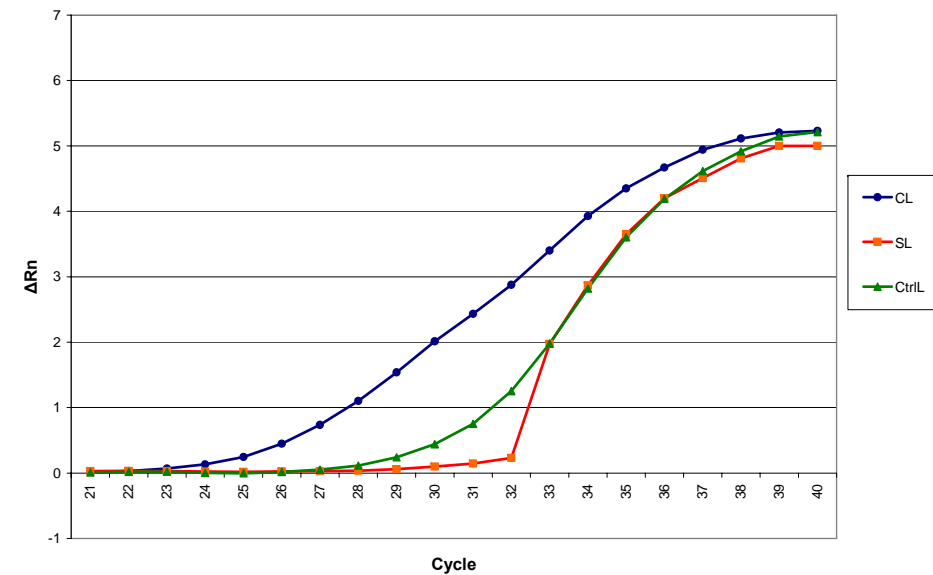

EST F379

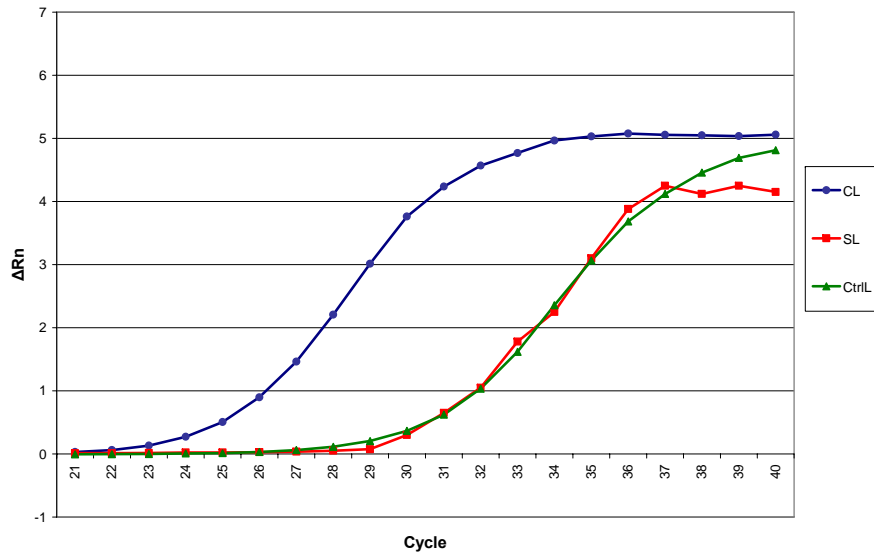

EST F443

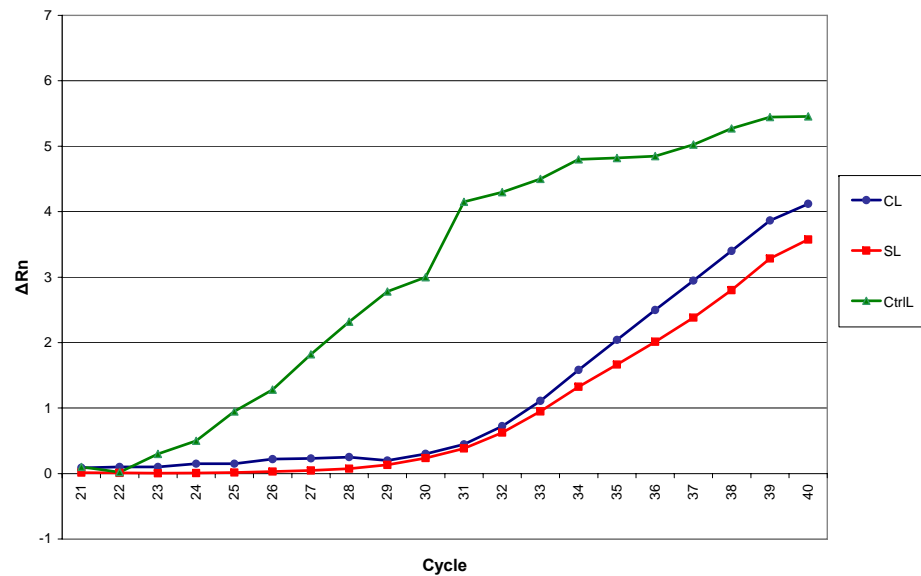

Supplement: Additional file 4 — qRT-PCR for differentially expressed candidate genes. Normalized report (ΔRn) vs. cycle for the ten candidate genes validated. CtrlL = control leaf, CL = cold leaf, SL: salinity leaf. Sunflower actin [GenBank: AAF82805) was used as reference "housekeeping" gene. Three biological samples were tested starting from the same RNA used as microarray hybridization probe. The average value for each of them was calculated and analysed in the graph. [file 1471-2229-8-11-S4.pdf]
